# Modelling the effects of variability in feeding rate on growth – a vital step for DEB-TKTD modelling

**DOI:** 10.1016/j.ecoenv.2022.113231

**Published:** 2022-03-01

**Authors:** Thomas Martin, Mark E. Hodson, Roman Ashauer

**Affiliations:** a University of York, Environment Department, Heslington, York YO10 5NG, UK; b Syngenta Crop Protection AG, Basel 4002, Switzerland

**Keywords:** ERA, Ecological Risk Assessment, MEM, Mechanistic Effect Model, TKTD, Toxicokinetic-Toxicodynamic, DEB, Dynamic Energy Budget, RMSE, Root Mean Square Error, SD, Standard Deviation, SE, Standard Error, Dynamic energy budget, Mechanistic modelling, Dietary toxicity, TK-TD

## Abstract

A major limitation of dietary toxicity studies on rodents is that food consumption often differs between treatments. The control treatment serves as a reference of how animals would have grown if not for the toxicant in their diet, but this comparison unavoidably conflates the effects of toxicity and feeding rate on body weight over time. A key advantage of toxicity models based on dynamic energy budget theory (DEB) is that chemical stress and food consumption are separate model inputs, so their effects on growth rate can be separated. To reduce data requirements, DEB convention is to derive a simplified feeding input, *f*, from food availability; its value ranges from zero (starvation) to one (food available *ad libitum*). Observed food consumption in dietary toxicity studies shows that, even in the control treatment, rats limit their food consumption, contradicting DEB assumptions regarding feeding rate. Relatively little work has focused on addressing this mismatch, but accurately modelling the effects of food intake on growth rate is essential for the effects of toxicity to be isolated. This can provide greater insight into the results of chronic toxicity studies and allows accurate extrapolation of toxic effects from laboratory data. Here we trial a new method for calculating *f*, based on the observed relationships between food consumption and body size in laboratory rats. We compare model results with those of the conventional DEB method and a previous effort to calculate *f* using observed food consumption data. Our results showed that the new method improved model accuracy while modelled reserve dynamics closely followed observed body fat percentage over time. The new method assumes that digestive efficiency increases with body size_._ Verifying this relationship through data collection would strengthen the basis of DEB theory and support the case for its use in ecological risk assessment.

## Highlights

• Models can separate a chemical’s direct toxic impact from effects on feeding rate.
• Feeding data can be used to construct high resolution inputs for growth models.
• This allows the use of simplified model equations to simulate mammal growth.
• A newly developed method for constructing feeding inputs improves model accuracy.
• This method also addresses theoretical issues associated with previous approaches.

## 1 Introduction

Mechanistic effects models (MEMs) aim to simulate the mechanisms by which chemicals affect individuals, populations and communities (Grimm and Martin, 2013). This is an appealing prospect with great potential for use in ecological risk assessment (ERA) of chemicals such as pesticides (Forbes et al., 2009, Forbes and Calow, 2012). Simulating underlying processes confers several advantages over traditional analysis of data from laboratory-based toxicity studies and extrapolations to field scenarios based on summary statistics. Mechanistic modelling enables the prediction of toxic effects in untested, ecologically relevant conditions. This can add ecological realism to extrapolations and potentially even reduce animal testing requirements (Jager et al., 2006).

Accounting for the mismatch in exposure between laboratory and field is a key obstacle to long term risk assessment of pesticides for mammals (Fischer, 2005). For example, in chronic toxicity testing of pesticides, rats are exposed to a constant concentration of test compound in their diet for up to two years (OECD, 2018a, OECD, 2018b, OECD, 2001). Such constant exposure is unrealistic in the field as pesticides are not applied at a constant rate all year round. This disparity can be addressed using toxicokinetic-toxicodynamic (TK-TD) models (Jager et al., 2006). These are a class of MEMs that work at the individual level, predicting an internal measure of chemical concentration over time (toxicokinetics) and the stress this places on an organism (toxicodynamics). As such, the effects on a given endpoint resulting from realistic, time varied exposure can be predicted (Nyman et al., 2012).

The use of TK-TD modelling has now been recommended for certain regulatory purposes, such as predicting survival of aquatic organisms (EFSA, 2018). However, for birds and mammals sublethal effects are most relevant at realistic exposure levels, as no mortality associated with pesticide use is accepted under European regulations (EFSA, 2009). The ‘DEBtox’ or ‘DEB-TKTD’ modelling framework (Kooijman and Bedaux, 1996b, Kooijman and Bedaux, 1996a, Sherborne et al., 2020), combining TK-TD modelling with the Dynamic Energy Budget (DEB) theory (Kooijman, 2009) provides a means of predicting sublethal toxic effects. DEB is an established metabolic theory, mathematically describing the processes of energy acquisition and allocation to predict endpoints such as body size and reproductive output. DEB has been applied to a wide range of taxa, with parameters available in the Add My Pet (AmP) library (Marques et al., 2018). The majority of DEB-TKTD studies thus far have focused on invertebrates (Ashauer and Jager, 2018) and more recently fish (Zimmer et al., 2018, Sadoul et al., 2018), with very few studies concerning terrestrial vertebrates (Martin et al., 2019, Desforges et al., 2017).

A particular advantage of DEB-TKTD modelling is the ability to separate the effects of feeding rate and direct toxic action on growth rate. This is particularly important in dietary toxicity studies, where ingested dose is directly related to feeding rate. This property is relatively unexplored but, in fact, it is crucial for TD models to accurately reflect toxicity and therefore to be of use for extrapolation to novel scenarios. Temporal and inter-treatment variability in feeding rate is a crucial driver of observed growth so any observed effects on body weight cannot simply be attributed to toxic action. Moreover, the extent to which a compound induces feeding avoidance may increase or decrease the risk posed to wildlife, depending on whether animals would have a choice of food items in the field scenario (Thompson, 2007). A previous study (Martin et al., 2019) developed methods to account for variability in feeding while modelling the effects of dietary toxicity on growth of domestic laboratory rats (*Rattus norvegicus*). However, some important issues with these methods were identified as areas for improvement in future.

In DEB theory, feeding rate is assumed to be limited by surface area (*e.g.* area of feeding appendages in filter feeders), which is proportional to body mass to the power 2/3. Where data are available, observed area specific feeding rate is divided by a maximum value so that it can be entered into the model as a dimensionless parameter ranging from zero to one (Jager et al., 2013, Kooijman, 2009). In Martin et al. (2019), we generated model inputs by scaling weekly area specific feeding rate in each treatment relative the maximum observed rate within each dataset. While this was a logical approach, two major issues became apparent.

The first issue was that, in rats, area specific feeding rate decreases as animals grow (Laaksonen et al., 2013, Martin et al., 2019). As such, the scaled feeding rate entered into the model dropped well below one in the latter stages of growth. This meant that, according to model equations, animals could have grown to many times their maximum observed body weight if they had continued to feed at the maximum observed rate throughout their lifetime. While this was a theoretical rather than a practical issue, it must be addressed for models to realistically represent the processes involved in growth.

The second issue arose because area specific feeding rate was calculated relative to observed (rather than predicted) body size. This meant that when predictions differed from observed data, this could result in a positive feedback loop or ‘snowball effect’. For example, if a rat with area of 40 cm^2^ ate 20 g food/day at time *t* this would be a feeding rate 0.5 g/cm^2^/day. However, if predicted surface area at time *t* were larger than that observed, say 50 cm^2^, then 0.5 g/cm^2^/day would equate to 25 g/day. Therefore, the modelled growth rate in the next time step would correspond to 25% higher food consumption than was observed, exacerbating the problem with each time step.

Here we investigate the potential of new methods to solve these issues and the implications for DEB theory. As suggested in Martin et al. (2019), we look to mathematically describe the relationship between feeding rate and body size in rats over the entire growth period and use this as a reference for scaling observed area specific feeding rate. We assess the resulting models from three standpoints: accuracy - how closely fitted models agreed with observed growth curves; generality – how well independent data are predicted without additional fitting; biological realism – how realistically the models simulate the processes underlying growth. We use a model based on the DEBkiss modelling framework (Jager et al., 2013) - a simplified version of DEB, following the same fundamental principles but with fewer parameters. It was desirable to prioritise model simplicity in this study. Firstly, because eliminating complex reserve dynamics from the model meant that the effects of different feeding inputs on model predictions could be more easily analysed. Data from studies in which no reproduction took place were also chosen for this reason. Additionally, the lack of user-friendly modelling tools was recently identified as a barrier preventing the use of DEB-TKTD models by regulators (EFSA, 2018) which has prompted renewed interest in DEBkiss (Jager, 2020).

## 2 Methods

### 2.1 Data

All data used here were made available from existing regulatory studies (Syngenta, unpublished) required under 94/79/EC (European Commission, 1994), investigating chronic toxicity of acibenzolar-S-methyl, prosulfuron and thiamethoxam in Sprague Dawley laboratory rats (*Rattus norvegicus*) (Palm, 1975).

Chronic toxicity studies lasting two years were carried out according to OECD guidelines (OECD, 2018a, OECD, 2018b, OECD, 2001). Animals were kept in standard conditions with food and water available *ad libitum*. Each study comprised a control group and at least three dose groups with individual observations of body weight (g) initially at weekly intervals (later observations were up to five weeks apart). Food consumption (g_(food)_ × day^−1^) was recorded alongside body weight either individually or per cage (2–5 individuals), providing the average per animal per day. Sample size was initially 80 animals per treatment per sex and only data for unmated animals were included in this study. Raw data used in this study are included in the Supporting Information.

### 2.2 Calibration dataset

The control group from the two-year dietary toxicity study of acibenzolar-S-methyl was selected as the calibration data in this study, as it was intermediate in terms of total food consumption for both sexes. This dataset, henceforth referred to as group A, comprised observations of an initial 80 animals of each sex at 37 timepoints over 104 weeks (a total of 2659 observations for males and 2678 for females). Animals in this study were fed a diet of Nafag 890 pelleted food.

### 2.3 Independent datasets

Independent datasets B and C were the control groups from the two-year dietary toxicity studies of prosulfuron and thiamethoxam respectively. Initial sample size in both datasets was 80 animals of each sex. Animals in group B were fed a diet of Rodent Chow #5002 pellets and observations took place at 37 timepoints over 104 weeks. Animals in group C were fed a diet of Nafag 890 pelleted food and observations took place at 36 timepoints over 103 weeks. Nafag 890 and Rodent Chow #5002 are similar in protein (18–20%), fat (3–4.5%) and energy content (12–14 kJ/g) although Nafag 890 is substantially higher in fibre (Ruhlen et al., 2011, Leonhardt and Langhans, 2002, Silberbauer et al., 2000).

### 2.4 Theoretical basis of the bioenergetic model

To simulate rat growth, we used a slightly modified version of the DEBkiss modelling framework (Jager et al., 2013). The model in this study employs the most basic rules for starvation and the storage of assimilates. The reason for this choice was to determine how accurately growth can be predicted using simple equations, if feeding data are used to produce accurate and high-resolution model inputs.

All DEB (Kooijman, 2009) models are based on the principle that certain processes are limited by volume or surface area and that an animal’s length, surface area and volume scale such that $\mathrm{Volume}\propto {\mathrm{Length}}^{3}$ and $\mathrm{Surface Area}\propto {\mathrm{Volume}}^{2/3}\propto {\mathrm{Length}}^{2}$, provided body shape remains the same (isomorphic growth). In DEBkiss (Jager et al., 2013), an animal’s total wet weight, $W_{w}$, is divided into structural (bones, muscle, organs *etc.*) weight, *W*_*V*_, and stored assimilates known as the reproduction buffer, $W_{R}$. DEBkiss was developed with invertebrates in mind and so the reproduction buffer is generally meant to provide mass for egg production. This function is clearly not applicable to viviparous mammals for which the cost of producing gametes is low. For female placental mammals, such as rats, the costs of reproduction occur during pregnancy and lactation. Data show that these costs are not paid for with an already accumulated buffer but are instead met in real-time, through increased food consumption (Bernard and Hohn, 1989, Morgan and Winick, 1981, Fontaine, 2012, Shirley, 1984). Therefore, we used an altered interpretation of the model equations, postulating that assimilates are stored simply to cover any maintenance costs that cannot be met by feeding in future. As such, it is more intuitive to think of $W_{R}$ simply as mass of ‘reserve’ and it will be referred to as such throughout. But for the notation and units, this implementation is very similar to the ‘DEBlipid’ model developed by Martin et al. (2017) and that of Desforges et al. (2017). Here, reserve simply refers to stored assimilates, primarily in the form of body fat, and so its definition differs from that given in full DEB models (Kooijman, 2009). DEBkiss assumes that juvenile animals allocate all available resources to growth and maturation, and so $W_{R}=0$ until the onset of puberty. From this point on a portion of assimilates are stored for reproductive investment (Jager et al., 2013). We make the same assumption, with the distinction that assimilates are stored to cover the costs of reproduction or maintenance as needed.

Wet weight is more practical to measure than structural volume, *V*. Assuming that average wet tissue density, $d_{w}$ (g × cm^−3^), is equal to that of water, that is $d_{w}$ = 1 (Lika et al., 2011), means that in juvenile animals ${V=W}_{w}/1$ g × cm^−3^. Rather than any specific measure of length, such as nose to tail, the volumetric length, *L*, is defined as *V*^1/3^ and surface area, *a*, is equal to *L*^2^ or *V*^2/3^_._ It is also helpful to estimate the density of structure, $d_{V}$ (g × cm^−3^), allowing conversion between dry weight and volume such that $W_{V}=V\times \;d_{V}$ and $W_{w}=d_{w}W_{V}/d_{V}+W_{R}$. Multiple studies have estimated average tissue water content of *R. norvegicus* as between 64% and 74% (Reinoso et al., 1997) suggesting that 0.3 is a realistic value of $d_{V}$ for this species.

### 2.5 Model notation

*J*: Flux or rate.
*y*: Yield or efficiency.
*d*: Density.
*X*: Food.
*A*: Assimilates.
*M*: Maintenance.
*R*: Reserve.
*W*: Weight or mass.
*V*: Structural volume.
*a*: Surface area.
*L*: Volumetric length.
*w*: Wet tissue.
*m*: Maximum.

### 2.6 Growth model

Assimilation of nutrients from food into the body occurs across membranes and so this process is assumed to be mediated by surface area. Assimilation flux, $J_{A}$*,* is defined as

(1) $$J_{A}=f{J_{\mathrm{Am}}^{a}V}^{\frac{2}{3}}$$

where $J_{\mathrm{Am}}^{a}$ is the maximum surface area specific assimilation rate (g_(assimilates)_ × cm_(*L*)_^−2^ × d^−1^) and *V* is volume. The parameter *f* is ‘scaled functional response’ to food availability (Jager et al., 2013, Kooijman et al., 2008) or ‘scaled feeding rate’ depending on how it is calculated. The distinction between these two terms is detailed later.

Maintenance flux, $J_{M}$*,* is given as

(2) $$J_{M}=J_{M}^{V}V$$

Where $J_{M}^{V}$ is the mass specific maintenance rate (g_(assimilates)_ × cm_(*L*)_^−3^ × d^−1^). Endotherms are also subject to surface area specific maintenance costs, accounting for heat loss to the environment. However as long as the ambient temperature is within the thermoneutral zone of a species (Kingma et al., 2014) these are assumed to be zero (Lika et al., 2011). Laboratory guidelines require rodents to be kept at 22 ± 3 °C, as this was considered to be within the thermoneutral zone of the rat (Poole and Stephenson, 1977). More recent research has suggested that this temperature range is too low (Le and Brown, 2008) but for simplicity we assumed that heat loss could be omitted.

It is assumed that a certain proportion of assimilates are allocated to structural maintenance and growth and this is denoted *κ* (dimensionless).

$\mathrm{If}\kappa J_{A}>J_{M}$, that is, assimilation is sufficient for growth and reserve storage

(3) $$\frac{dW_{V}}{\mathrm{dt}}=y_{\mathrm{VA}}(\kappa J_{A}-J_{M})$$

(4) $$\frac{dW_{R}}{\mathrm{dt}}=(1-\kappa )J_{A}$$

**Fig. 1:**
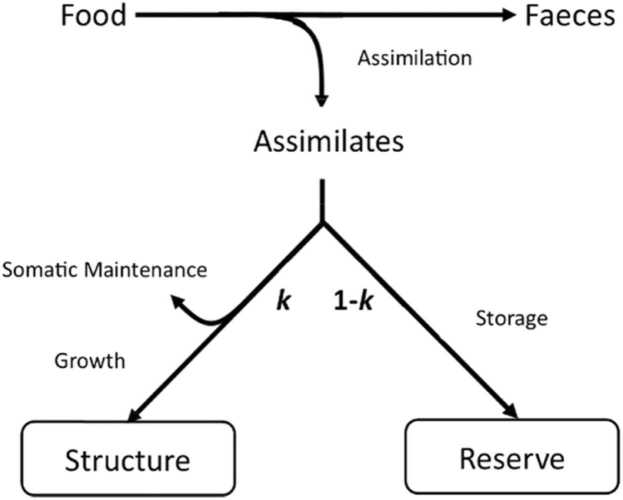
A graphical representation of the growth model when assimilation is sufficient for growth. The value of *κ* determines the proportion of resources assimilated from food allocated to maintenance and growth or stored as reserve.

Where $y_{\mathrm{VA}}$ (g_(structure)_ × g_(assimilates)_^−1^) is the yield of structure over assimilates, (*i.e.* the efficiency with which assimilates can be converted into structure). Puberty is estimated to begin in rats at 5–7 weeks of age (Rakel and Gergs, 2018) which is also the age of the study animals at the start of observation. As such, we assumed that $W_{R}=0$ initially and begins to accumulate immediately. Like the full DEB model, our model implementation divides wet weight into structure and reserve (although reserve is more narrowly defined in this case). However, the model equations used are unaltered from DEBkiss and follow the simple assumption that any assimilates not required for maintenance, or allocated to growth, are stored. This system is represented in Fig. 1.

At any constant value of *f*, growth ceases when $J_{A}=J_{M}$. This is the point at which the ultimate structural volume, $V_{\infty }$, is reached, which can be calculated as ${\left({\kappa \mathrm{fJ}}_{\mathrm{Am}}^{a}/J_{M}^{v}\right)}^{3}$. The theoretical maximum structural volume, $V_{m}$, is reached when $J_{A}=J_{M}$ and *f* = 1 such that $V_{m}={\left({\kappa J}_{\mathrm{Am}}^{a}/J_{M}^{V}\right)}^{3}$. At all times $\frac{dW_{w}}{\mathrm{dt}}=\frac{d_{w}}{d_{V}}\;\frac{dW_{V}}{\mathrm{dt}}+\frac{dW_{R}}{\mathrm{dt}}$ but $\frac{dW_{V}}{\mathrm{dt}}$ and $\frac{dW_{V}}{\mathrm{dt}}$ depend on the value of *f*.

If $\kappa J_{A}<J_{M}<J_{A}$, that is, overall assimilation flux *JA* is sufficient for homeostasis but not growth then

(5) $$\frac{dW_{V}}{\mathrm{dt}}=0$$

(6) $$\frac{dW_{R}}{\mathrm{dt}}=J_{A}-J_{M}$$

Maintenance is prioritised above growth, with the 1 - *κ* branch utilised to pay maintenance costs and any remainder stored as reserve. (5), (6) also describe change in body mass when $J_{A}<J_{M}$ and $W_{R}>0$. In this scenario, the animal is starving, and *W*_*V*_ is maintained by utilising reserve. Both $\frac{dW_{R}}{\mathrm{dt}}$ and $\frac{dW_{w}}{\mathrm{dt}}$ become negative as the reserve decreases.

If $J_{A}<J_{M}$ and $W_{R}=0$, that is, reserve has been used up and assimilation is insufficient to meet maintenance costs

(7) $$\frac{dW_{V}}{\mathrm{dt}}=(J_{A}-J_{M})/y_{\mathrm{AV}}$$

(8) $$\frac{dW_{R}}{\mathrm{dt}}=0$$

where $y_{\mathrm{AV}}$ (g_(assimilates)_ × g_(structure)_^−1^) is the yield of assimilates over structure (*i.e.* the efficiency with which assimilates can be extracted from structure). Therefore, structural weight is lost until it can be sustained by feeding.

In order to minimise the number of free parameters, the values of several parameters were fixed. The parameters $y_{\mathrm{AV}}$ and $y_{\mathrm{VA}}$ were both assigned their default value of 0.80 (Jager et al., 2013). The parameter *κ* is generally estimated using data for body size and reproduction over time (Kooijman et al., 2008). This was not possible with our calibration dataset, as the animals did not reproduce. Length data, which would have allowed differentiation between growth and weight gain as fat, were also unavailable. Since *κ* could not be estimated, we used the value of 0.9472, taken from the most recent AmP entry for *R. norvegicus* (Rakel and Gergs, 2018).

The maximum surface area specific assimilation rate, $J_{\mathrm{Am}}^{a}$ and volume specific maintenance rate, $J_{M}^{V}$, were fitted to data. The value of *f* was calculated from data or food availability. Various approaches to this calculation, and their theoretical implications, are now summarised.

#### 2.6.1 Method 1: *f* = scaled functional response to food availability

The approach used most commonly in DEB literature is to calculate the value of *f* based on food availability because, in most cases, detailed feeding data are unavailable. This approach uses the Holling Type II functional response

(9) f=X/(X+H)

where *X* denotes the density of food in the environment (g_*(food)*_ × m^−2^) and *H* (g_*(food)*_ × m^−2^) is the half-saturation food density at which food consumption rate, $J_{X}$ (g_*(food)*_ × day^−1^) is half of its maximum. When food is available *ad libitum,* X=∞ and therefore f=1 (Van Der Meer, 2006, Kooijman et al., 2008, Jager et al., 2013). Using food availability as a proxy for observations of food consumption in this way relies on the assumption that, when provided with as much food as they can eat, animals eat as much as they can.

In other studies, *f* has been fixed to one during calibration and then estimated for independent data (Sadoul et al., 2018). This approach was not followed in this study as it is based on observed growth rather than feeding data, so the mechanistic basis is unclear. An alternative would be to compare overall average area specific feeding rate in the independent dataset to that of the calibration dataset and adjust *f* accordingly. However, this would not test whether food availability is a suitable proxy for feeding observations. Since feeding availability was always *ad libitum* in all datasets included in this study, *f* was fixed at 1 for all datasets in this study.

#### 2.6.2 Methods 2 & 3: from scaled functional response to scaled feeding rate

The rate at which an animal can consume food depends on body size and so $J_{X}$ has no fixed upper limit. Instead, it is assumed that feeding rate is limited by surface area so

(10) $$J_{X}=fJ_{\mathrm{Xm}}^{a}L^{2}$$

where $J_{\mathrm{Xm}}^{a}\;$(g_*(food)*_ × cm_(*L*)_^−2^ × day^−1^) is the maximum area specific feeding rate for a species and L is the animal’s volumetric length (cm) (Jager et al., 2013).

(11) $$J_{\mathrm{Xm}}^{a}=J_{\mathrm{Am}}^{a}/y_{\mathrm{AX}}$$

where $y_{\mathrm{AX}}$ is the yield of assimilates from food or digestive efficiency (g_*(assimilates)*_ × g_*(food)*_^−1^) and $J_{\mathrm{Am}}^{a}$ is the maximum surface area specific assimilation rate (g_(*assimilates*)_ × cm_(*L*)_^−2^ × d^−1^). Since $y_{\mathrm{AX}}\leq 1$, $J_{\mathrm{Xm}}^{a}$ provides the upper limit when fitting $J_{\mathrm{Am}}^{a}$.

Dividing Eq. 10 by $L^{2}$gives

(12) $$J_{X}^{a}=fJ_{\mathrm{Xm}}^{a}$$

where $J_{X}^{a}$ is area specific feeding rate (g_(*food*)_ × cm_(*L*)_^−2^ × day^−1^). Solving for *f* gives

(13) $${f=\;J}_{X}^{a\;}/{\;J}_{\mathrm{Xm}}^{a}$$

So, where $J_{X}^{a}$ can be calculated from observed data, it is more appropriate to calculate *f* using Eq. 13 and define it as ‘scaled feeding rate’ rather than scaled functional response. In Methods 2 and 3, $J_{X}^{a}$ is calculated for each observation interval by dividing observed of daily food consumption by the associated observation of wet weight, $W_{w}$, raised to the power of 2/3. Strictly speaking, calculations of $J_{X}^{a}$ should be based on structural surface area, *a* or *V*^2/3^. However, since *V* is not quantifiable from observed data, $W_{w}$ was used instead. While both Methods 2 and 3 use Eq. 13, they differ in how $J_{\mathrm{Xm}}^{a}$ is calculated.

#### 2.6.3 Method 2: $J_{\mathrm{Xm}}^{a}$ = maximum observed area specific feeding rate

In this approach, previously employed in Martin et al. (2019), $J_{\mathrm{Xm}}^{a}$ is defined as the maximum individual observed area specific feeding rate within a dataset (separated by sex). In group A, $J_{\mathrm{Xm}}^{a}$ was 0.822 g × cm^−2^ × day^−1^ for males and 0.715 g × cm^−2^ × day^−1^ for females. These values were used for all datasets. Identifying $J_{\mathrm{Xm}}^{a}$ in this way guarantees that scaled *f* values do not exceed one for the calibration data set. It is possible, though unlikely, for *f* to exceed one when using (mean) independent data.

#### 2.6.4 Method 3: $J_{\mathrm{Xm}}^{a}$ = predicted $J_{X}^{a}$ at a given body size, maximum food availability

In this method, observed daily food consumption, $J_{X}$ (g_*(food)*_ × day^−1^), and area specific feeding rate, $J_{X}^{a}$ (g_*(food)*_ × cm_(*L*)_^−2^ × day^−1^) with food available *ad libitum*, were described empirically as functions of surface area (calculated as ${\left(W_{w}/d_{w}\right)}^{2/3}$). Visual inspection showed that, rather than continually increasing as animals grew, $J_{X}$ roughly followed a sigmoid pattern when plotted against surface area. The generalised logistic function (Richards, 1959) was selected as a flexible sigmoid curve which could meet the necessary conditions to model $J_{X}$ as a function of body size. It was specified that the curve must pass through the origin, as an animal with zero mass would be unable to consume any food.

One expression of the generalised logistic formula to describe *J*_*X*_ in terms of surface area, *a*, is

(14) $$J_{X}=G+\;\frac{U-G}{{1+e}^{-B(a-M)}}$$

Where *G* is the lower asymptote (g_(*food*)_ × day^−1^), *U* is the upper asymptote (g_(*food*)_ × day^−1^), *M* is inflection point (cm^2^) and B is the growth rate (cm^−2^). The simplest way (minimum number of parameters) in which this can be adjusted to pass through the origin is as a symmetrical curve with its inflection point at (0,0). This can be done by stipulating that G=−U and M=0 such that

(15) $$J_{X}=\frac{2U}{1+e^{-B(a)}}-U$$

**Fig. 2:**
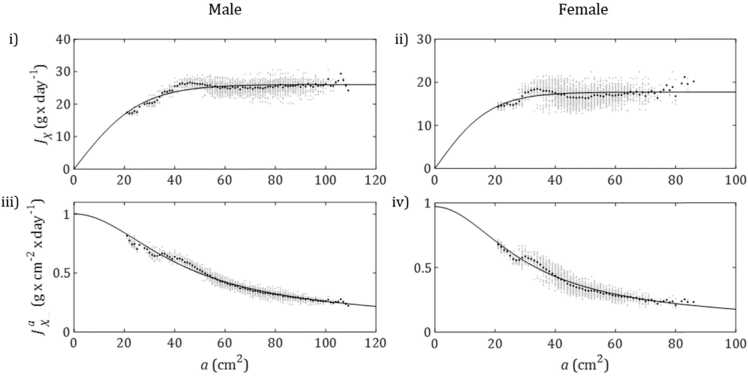
Plots i) & ii) show observed (circles) and modelled (line) daily food consumption $J_{X}$*vs* surface *a*rea, *a*, of males and females respectively. Raw data are plotted in light grey while mean values are plotted in black. Method 3 uses the formula $J_{X}=\frac{2U}{1+e^{-B(a)}}-U$, fitted to mean data to model food consumption per day. For males *U*= 26.03 and *B*= 0.07693 while for females *U*= 17.72 and *B*= 0.1096. Plots iii) & iv) show observed (circles) and modelled (line) area specific feeding rate $J_{X}^{a}$*vs* surface area, *a*, of males and females respectively. Raw data are plotted in light grey while mean values are plotted in black. Models plot the fitted formula for $J_{X}$ divided by surface area, *a*.

With only two free parameters, *U* and *B*, this function was then fitted to mean $J_{X}$ (at each unique value of *a*) in the calibration dataset (Fig. 2). The coefficient of determination, *R*^2^, was then calculated. This showed that, for males, 82% of variation in mean $J_{X}$ was explained by the fitted function of surface area. For females, this figure was 38%. In order to eliminate values of $J_{X}$ that were insufficient to meet maintenance costs, any data collected after mean body size had peaked (day 539 for males, day 686 for females) were excluded. Area specific feeding rate, $J_{X}^{a}$, was then simply modelled as

(16) $$J_{X}^{a}=\;J_{X}/a$$

This explained 98% of variability in mean area specific feeding rate for males and females (Fig. 2 iii-iv). Modelled $J_{X}^{a}$, as a function of *a*, was then used as the reference for scaling observed $J_{X}^{a}$, meaning that $J_{\mathrm{Xm}}^{a}$ was redefined as predicted area specific feeding rate at a given body size, at maximum food availability (Eq. 16).

Redefining $J_{\mathrm{Xm}}^{a}$ has several important theoretical implications. Because $J_{\mathrm{Xm}}^{a}$ is no longer a true maximum, the scaled feeding rate, *f*, may exceed one. Consequently, $J_{\mathrm{Am}}^{a}$ no longer represents an absolute maximum either and is redefined as the area specific assimilation rate predicted at maximum food availability. Furthermore, its upper limit must be the lowest predicted $J_{\mathrm{Xm}}^{a}$ within the observed range of body size (Eq. 11). Crucially, as per Eq. 11, if $J_{\mathrm{Am}}^{a}$ remains fixed but $J_{\mathrm{Xm}}^{a}$ decreases as animals grow, then digestive efficiency,$\;y_{\mathrm{AX}}$, must increase with body size.

### 2.7 Addressing the ‘snowball effect’ in Methods 2 and 3

To avoid the feedback loop described in the introduction (Martin et al., 2019), $J_{X}^{a}$ was calculated in real time from observed food consumption, $J_{X}$ (g × day ^−1^), and modelled surface area, *a* (cm^2^). Surface area, *a*, at time *t* was defined as modelled ${\left(W_{w}/d_{w}\right)}^{2/3}$ (in order to be consistent with how $J_{X}^{a}$ was calculated from data). Mean observed food consumption, $J_{X}$, at time *t* was then divided by *a*, to yield $J_{X}^{a}$ for the next time step. In Method 2, $J_{X}^{a}$ was simply divided by the fixed value of $J_{\mathrm{Xm}}^{a}$ to give a value of f at time *t*. In Method 3, $J_{\mathrm{Xm}}^{a}$ was calculated by entering modelled *a* at time *t* into (15), (16) before using Eq. 13 to yield *f*. This meant that growth rate was modelled based on observed food consumption rather than observed area specific feeding rate over time.

### 2.8 Model assessment

Initially, the growth model was fitted to wet weight data from the calibration dataset A using each method of *f* calculation. To reduce the impact of heteroscedasticity, the square root transformation was used during fitting. The accuracy of the model fits was then assessed in a variety of ways. Overall goodness of fit was measured with the coefficient of determination, *R*^2^, and the root mean square error, RMSE. Additionally, the proportion of observations predicted to within one standard deviation of the mean was calculated.

Next, the models were used to predict independent datasets B and C, without recalibration, to assess the generality of the model parameters derived using each method. Again, predictions were assessed using *R*^2^, RMSE and the proportion of observations predicted to within one standard deviation of the mean.

The biological realism of each method was then assessed through comparison of the theoretical maximum volume, $V_{m}$, and modelled reserve, $W_{R}$, to relevant literature data. Finally, the impact of real time *f* calculation to avoid positive feedback was assessed with a worked example.

### 2.9 Model implementation

All models were implemented in Matlab (ver. R2020a). Growth models were developed with the BYOM (Jager, 2019) flexible model platform (ver. 4.1). All fitted parameter values were derived using the Nelder Mead simplex algorithm to maximise the likelihood function, given the observed data (Pan and Fang, 2002). Likelihood profiling was also used to check that initial fits were not local optima (Kreutz et al., 2013).

## 3 Results

### 3.1 Calibrated growth curves

**Fig. 3:**
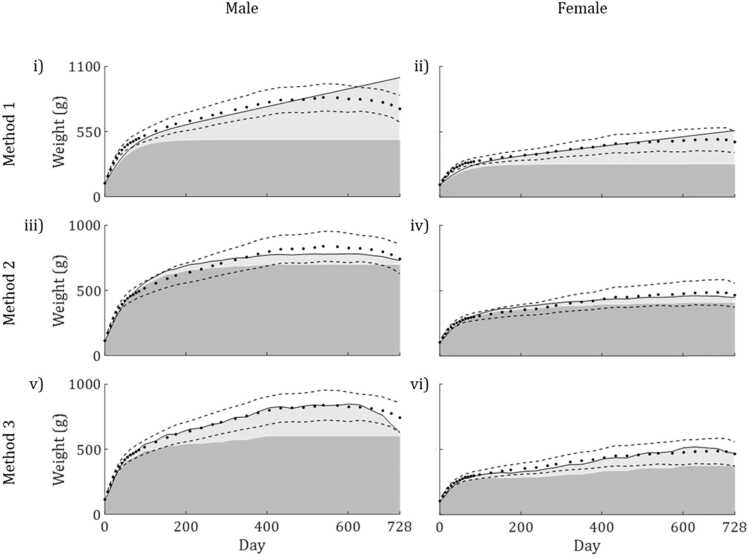
Plots showing models (solid line) fitted to observed mean body weight of group A male and female rats over 2 years (circles). The shaded area under the model curves shows structure (dark grey) and reserve (light grey) while dashed lines represent observed mean ± SD. The results of Method 1 are shown in plots i-ii, Method 2 in plots iii-iv, and Method 3 in plot v-vi.

The growth curve was fitted to mean wet weight, $W_{w}$, observed over two years in group A, using each of the three methods for determining the scaled feeding rate, *f* (Fig. 3). Fitted parameter values as well as various measures to assess goodness of fit are given in Table 1. Goodness of fit measures only relate to total body weight, $W_{w}$, as this was the only model variable monitored in the dietary toxicity studies which provided data for this investigation. For illustrative purposes, the breakdown of modelled $W_{w}$ into reserve and structure is shown on plots. Modelled reserve dynamics are assessed with respect to literature data in the Biological Realism subsection.

**Table 1:** Fitted parameter values, selected observed and modelled endpoints, and goodness of fit measures for each method of calculating the scaled feeding rate, f.

Sex	Males			Females		
Observed max. $W_{w}$ (g)	839.0			486.2		
Method	1	2	3	1	2	3
$J_{\mathrm{AM}}^{a}$ (g × cm^−2^ × d^−1^)	0.2310	0.1186	0.1919	0.1764	0.07970	0.1317
$J_{M}^{V}$ (g × cm^−3^ × d^−1^)	0.02791	0.004798	0.02181	0.02557	0.004315	0.01762
Modelled max. $W_{w}$ (g)	1006.6	779.6	854.9	559.4	459.8	515.6
R^2^	0.857	0.970	0.985	0.900	0.967	0.973
RMSE	79.52	36.54	25.83	33.21	19.33	17.53
% $W_{w}$ observations modelled to within 1 SD	61.11	86.11	91.67	66.67	86.11	91.67

In Method 1, *f* = 1 for the duration of the study. This meant that a smooth curve was produced and stored reserve, $W_{R}$, rose continuously. Consequently, this method produced the weakest fits to mean body weight, $W_{w}$, over time. For both sexes, this method produced the lowest R^2^, the highest RMSE and modelled the fewest observations to within 1 sd. of the mean.

Method 2 defined $J_{\mathrm{Xm}}^{a}$ as the highest observed area specific feeding rate in group A. Good fits were calculated for mean $W_{w}$ over time (*R*^2^ > 0.96). Modelled growth rate fluctuated in response to variation in food intake over time and became negative toward the end of the study period, matching observations. The overall shape of the curve was similar for males and females, with signs that the error, though small, was systematic. Modelled growth rate lagged behind that observed until modelled body weight overtook observations after around 60–75 days. This persisted until modelled body weight fell below observations once again after 350–400 days (Fig. 3 iii-iv).

In Method 3, $J_{\mathrm{Xm}}^{a}$ was calculated as a function of surface area, *a*. The calculated fits to mean data were slightly better, for all measures, than those of Method 2. Modelled growth rate was highly responsive to fluctuations in *f*, becoming negative as area specific feeding rate dropped in the late stages. For both sexes, modelled body weights were very close to observed data for most of the observation period, with significant deviations only occurring late in the study. Maximum modelled $W_{w}$ was only 2% higher than the maximum observed body weight in males and 6% higher for females.

### 3.2 Summary analysis of food consumption and body weight data

Based on mean observed body weight and food consumption at each timepoint, summary analyses were conducted to highlight broad differences between the data sets (see supporting table S4). For both males and females, total food consumption was highest in group B, intermediate in group A and lowest in group C. Males and females in group A had the lowest starting weight but were intermediate in terms of maximum body weight and final body weight, with the highest weight gain (final weight minus initial weight) over two years. Weight gain, maximum weight and final weight were lowest for males and females in group C.

### 3.3 Feeding rate predictions

As part of Method 3, the generalised logistic curve was fitted to mean observed daily feeding rate, $J_{X}$ (g × day^−1^), as a function of surface area, *a*, (cm^2^) of male and female rats in group A. This produced R^2^ values of 0.82 and 0.37 respectively. Dividing fitted $J_{X}$ by *a* to predict, mean area specific feeding rate at maximum food availability, $J_{X}^{a}$, produced R^2^ values of 0.98 for males and females.

To assess the uniformity of the relationship between $J_{X}^{a}$ and *a* across study groups, the predictions of the calibrated curves were compared to independent datasets B and C. For males, variation in mean $J_{X}^{a}$ was well predicted by surface area with *R*^2^ values of 0.90 for group B and 0.95 for group C. Observed $J_{X}^{a}$ in group B agreed closely with predictions at medium body sizes but exceeded predictions at large sizes and showed a decrease at low body size that was not evident in the calibration data. Observed $J_{X}^{a}$ in group C showed a similar shape to the predicted curve but was generally slightly lower. The relationship was less consistent for females though, R^2^ was 0.68 for group B and 0.95 for group C. Observed $J_{X}^{a}$ in group B was higher than predicted, particularly at larger body sizes. As was the case for males, $J_{X}^{a}$ in group C was slightly lower than predicted for most body sizes.

The relationship between $J_{X}^{a}$ and *a* appears less uniform among female rats. However, deviations from predicted $J_{X}^{a}$ may be reflected by a predictable increase or decrease in growth rate. These results are shown in supporting Fig. S37.

### 3.4 Growth curve validation

The calibrated growth models were used to predict independent datasets B and C. The accuracy of the predictions produced by each method was assessed by calculating R^2^, RMSE and the percentage of observations predicted to within one standard deviation (Table 2).

**Table 2:** Selected measures of the accuracy of each method when used to predict independent data.

Sex	Male					
Dataset	B			C		
Method	1	2	3	1	2	3
R^2^	0.7753	0.9621	0.9452	0.0875	0.7430	0.8623
RMSE	86.16	35.38	42.54	137.8	73.15	53.54
% observations predicted to ± 1 sd.	36.11	86.11	80.56	34.29	60	80.00
Sex	Female					
Dataset	B			C		
Method	1	2	3	1	2	3
R^2^	0.8830	0.9254	0.2824	0.5537	0.8029	0.6229
RMSE	40.27	32.15	99.72	54.40	36.15	50.01
% observations predicted to ± 1 sd.	86.11	75.00	16.67	71.43	71.43	74.29

Method 1 produced virtually identical curves for all datasets (supporting Figs. S7-S10 & S25-S28). This is because initial weight was the only model input that differed from the calibration data. This method produced the poorest predictions of mean body weight over time for males in both independent datasets and for females in group C. For females in group B, Method 1 produced the highest proportion (86.11%) of predictions within one standard deviation of the observed mean. However, had the model continued to run, modelled body weight would have continued to increase, as reserve accumulated indefinitely.

Method 2 (supporting Figs. S11-S13 & S29-S32) produced the most accurate predictions (highest *R*^2^ and lowest MRSE) of mean growth rate for males and females in group B. Body weight of both sexes in group C was overpredicted for all but the early stages of observation. Despite this, Method 2 did produce the most accurate predictions for females in this dataset.

**Fig. 4:**
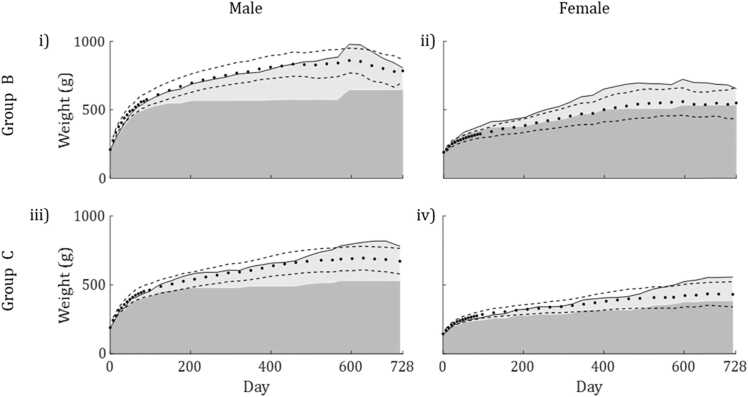
Plots showing predicted (solid line) and mean observed body weight of rats over 2 years (circles), using Method 3 to calculate the scaled feeding rate, *f*. The shaded area under the model curves shows structure (dark grey) and reserve (light grey) while dashed lines represent observed mean ± SD. The results for males and females in group B are shown in plots i-ii respectively while the results for males and females in group C are shown in plots iii-iv respectively.

Method 3 predicted growth of males in group B only slightly less accurately than Method 2, and was the most accurate for males in group C. In both cases, model predictions closely followed the observations until the later stages of observation. This was also the case for females in group C, for which this method produced the second most accurate predictions. However, growth of females in group B was poorly predicted. Modelled body weight was well above that observed for almost all of the observation period. These predictions are shown in Fig. 4.

### 3.5 Biological realism

Although goodness of fit to observed $W_{w}$ (the only model endpoint measured in toxicity studies) quantifies model accuracy, it gives no information as to the biological realism of the model itself. In order to address this question, literature data were utilised to assess other model variables. No data are available for $W_{R}$, as this represents stored assimilates from food. This would include not only stored lipids but also carbohydrates stored as glycogen, and fat-soluble vitamins. Nevertheless, observed body fat percentage of *ad libitum* fed rats is a useful, if not ideal, comparator, as it would be expected to follow very similar temporal patterns.

**Fig. 5:**
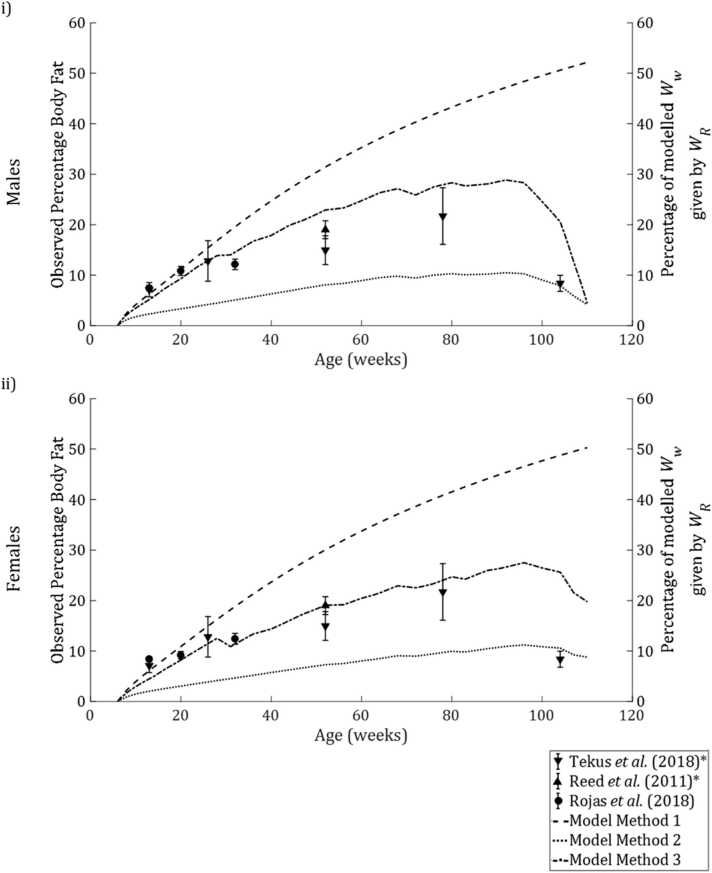
Plots showing mean ± SE body fat percentage (left hand axis) recorded in rats of various ages by Tekus et al. (2018), Reed et al. (2011) and Rojas et al. (2018), and calibrated model simulations of $W_{R}$ as a percentage of $W_{w}$ over time (right hand axis) for male (plot i) and female rats (plot ii). * denotes that data were available for male animals only.

Data from Tekus et al. (2018) provide reference values of body fat percentage of rats at various ages up to two years. While the study used only male Wistar (rather than Sprague Dawley) rats, other studies indicate that body fat percentage is similar across the two strains (Reed et al., 2011) and between male and female Sprague Dawley rats (Rojas et al., 2018). Fig. 5 shows literature data plotted against calibrated model simulations (group A) of $W_{R}$ as a percentage of modelled $W_{w}$.

Method 1 assumes that *f* = 1 at all times where food is available *ad libitum*, leading to constant accumulation of reserve. For both sexes, modelled $W_{R}$ was over 50% of modelled $W_{w}$ after 2 years, over five times the value reported at that age and more than double the maximum reported percentage body fat. Using Method 2, the observed decline in body fat in the late life stages was reflected by model simulations. $W_{R}$ peaked at 10.46% of modelled $W_{w}$ of males and 11.15% for females, only about half of the value reported by Tekus et al. (2018).

With Method 3, model simulations were relatively consistent with observations. For both sexes, $W_{R}$ as a percentage of $W_{w}$ matched observed body fat percentage at 26 weeks of age before reaching a peak between the ages of 78 and 104 weeks and declining thereafter. Peak $W_{R}$ percentage was 29.71% for males and 27.54%, slightly exceeding the highest mean body fat percentage + standard error (27.33%) reported by Tekus et al. (2018).

The maximum volume of structure,$\;V_{m}$, is a theoretical maximum calculated from model parameters as ${\left({\kappa J}_{\mathrm{Am}}^{a}/J_{M}^{v}\right)}^{3}$. Multiplying$\;V_{m}$ by the density of wet tissue, $d_{w}$, (assumed to be 1 g × cm^−3^ (Lika et al., 2011)) gives the maximum wet weight of structure, $W_{\mathrm{Vmw}}$. Peak lean weight would serve as a sensible proxy for comparison but is not measured in toxicity studies. Instead, we can assume that the weights of structure and reserve peak simultaneously, meaning that $W_{\mathrm{Vmw}}$ can be estimated from data as

(17) $$W_{\mathrm{Vmw}}=\max.\mathrm{observed}\;\mathrm{body}\;\mathrm{weight}\;\times (1-\max.\mathrm{observed}\;\mathrm{proportion}\;\mathrm{body}\;\mathrm{fat})$$

Using the relevant values from group A (used in calibration) and Tekus et al., gives 839.04 g × 0.783 = 656.97 g for males and 486.21 g × 0.783 = 380.70 g for females.

Modelled $W_{\mathrm{Vmw}}$ was lowest when using Method 1, for both sexes its value was 73% of that estimated from data. Method 2 meanwhile produced very high values of $W_{\mathrm{Vmw}}$, almost 20 times the estimated value for males and over 14 times the estimate for females. The values of $W_{\mathrm{Vwm}}$ given by Method 3 were 579.1 g for males and 356.5 g for females, 88% and 93% of the respective estimates for each sex. This was also the only method for which $W_{\mathrm{Vmw}}$ was not a strict maximum, as *f* could exceed one. The highest modelled wet weights of structure using this method were closer still at 601.0 g for males and 373.6 g for females. These results are summarised in supporting table S5.

### 3.6 Impact of real time *f* calculations

In this study, the value of *f* was calculated in real time to ensure that it reflected the quantity of food consumed rather than the observed area specific feeding rate at a given time point. This only applied to Methods 2 & 3, as Method 1 was based on food availability only. A worked example was conducted (included in Supporting Information) which demonstrated the effect of this new approach on growth rate predictions. This showed that calculating *f* in real time worked effectively to curtail the ‘snowball effect’ identified in Martin et al. (2019) and that this would otherwise have been a major issue for Method 2.

## 4 Discussion

DEB models are designed to function without the need for detailed feeding data (Kooijman, 2009, Jager et al., 2013). However, this presents the question of what to do with such data when they are available. The conventional approach to deriving feeding inputs in DEB models does not reflect temporal or intertreatment variability in feeding rate, only food availability. In a previous study (Martin et al., 2019), we developed a method to derive feeding inputs directly from feeding data, but this approach had problems of its own. In this study, a novel method was developed, with the aim of addressing all the issues previously identified. We used a simple model based on DEBkiss to assess three approaches, for their impact on model accuracy, generality and realism.

### 4.1 Accuracy and generality

Model accuracy was assessed by fitting the models to growth data for Group A. Method 3 produced the most accurate fits to calibration data for both males and females. Method 2 was only slightly less accurate. However, errors appeared more systematic in nature when using Method 2, following a similar pattern over time for both males and females. Method 1 was the least accurate, producing a smooth curve which did not respond to temporal variability in area specific feeding rate.

Model generality was then assessed by using the models to predict independent growth data (Groups B and C) without recalibration. Method 2 performed best in terms of model generality, despite systematic errors still being apparent. Without recalibration, body weight over time was predicted most accurately using this method for females in both independent datasets and for males in group B. Method 1 was again least accurate for all but one dataset. Using this method, the only model input to change between datasets was initial body weight which has minimal effects on predictions. The resulting model outputs for Method 1 were essentially different sections of the same curve for all datasets. While body weight of group B females was predicted relatively well, this result was coincidental as this dataset was quite different to that used in the calibration.

Predictions using Method 3 were most accurate for group C males and a close second for males in group B. Results were mixed for females however, this method was second most accurate for group C females but the least accurate for those in group B. It is notable that observed feeding patterns in this dataset were most different from predictions of $J_{\mathrm{Xm}}^{a}$ and that animals were fed on a different diet to those in groups A and C. While predictions of total body weight of group B females were poor, it is notable that predicted structural weight followed observed wet weight very closely (Fig. 4). This suggests that the profile of the scaled feeding rate, *f*, over time was accurate, if not the values themselves.

### 4.2 Addressing the ‘snowball effect’

Real time calculation of *f* works effectively to stop the ‘snowball effect’ that occurred in a previous study. Our worked example (see Supporting Information) showed that this step made a large difference to predicted ${∆W}_{w}$ when using Method 2. This calls into question some of the findings of Martin et al. (2019), in which feeding inputs were calculated before the models were run. In that study the relative contributions of feeding and toxicity toward observed effects on body weight over time were estimated. In most cases, positive feedback between predicted body size and predicted growth rate would have meant that the effects of feeding avoidance were understated. Though Method 3 mitigated the problem, it should still be considered good practice in future studies to calculate *f* as the model runs, especially when predicting independent data.

### 4.3 Biological realism

Method 1, that is suggested by DEB literature (Kooijman et al., 2008, Van Der Meer, 2006, Jager et al., 2013), assumes that *f* = 1 when food is freely available. In our growth model, this meant that $W_{\mathrm{Vmw}}$ was approached quickly with ${∆W}_{R}$ becoming linear *ad infinitum*. Modelled $W_{R}$ reached over 50% of $W_{w}$ after 2 years, more than twice the maximum reported percentage body fat in the literature.

It should be mentioned that our model did not include the maturity maintenance parameter, this represents the costs of maintaining sexual maturity and is taken from the 1- *κ* branch of the model. However, these costs are assumed to be proportional to structural weight at puberty and so do not increase with body size beyond that point (Jager et al., 2013). Therefore, maturity maintenance would have only slightly mitigated the issue of constant reserve accumulation. A potential solution would be to implement the full DEB model which uses a more complex equation to model reserve dynamics. This includes an additional parameter, the maximum reserve density (J_(*reserve*)_ × cm^−3^_(*structure*)_), which provides a limit on reserve accumulation. However, despite using the same simple equations, constant reserve accumulation was not an issue with Methods 2 and 3. It may then be the case that, rather than representing a real biological limit, the maximum reserve density simply serves to compensate for the oversimplicity of the feeding input.

However, the major issue with Method 1 is that rats provided with food *ad libitum* do not feed at the maximum area specific rate, as is assumed (see supporting Fig. S38). Kooijman (2009) acknowledged previously that rats do not conform to DEB feeding assumptions and instead modelled growth data from Hubert et al. (2000) by linking daily food consumption to the probability of enzymes binding to substrate. However, this method assumed that rats ate a fixed amount of food per day regardless of size (this is how data were summarised in the original paper) despite substantial temporal variability being evident. Rats regulate their feeding significantly as they grow (Laaksonen et al., 2013, Martin et al., 2019, Hubert et al., 2000), food consumption initially increases with body size before plateauing and declining in old age. In fact, data in this study and others (Hubert et al., 2000, Tekus et al., 2018) show that weight loss occurs as rats approach two years of age. These patterns cannot possibly be modelled based on constant *ad libitum* food availability (Eq. 9). Therefore, *f* ‡ 1 and/or digestive efficiency, $y_{\mathrm{AX}}$, and maximum area specific feeding rate,$\;J_{\mathrm{Xm}}^{a}$, are not fixed values.

While reproduction was not modelled in this study, it also seems logical that the value of *f* must vary with feeding data in order to accurately model body weight change during pregnancy and lactation. Placental mammals do not constantly amass a buffer in preparation for reproduction, as DEB theory generally assumes. Instead, studies show that females of diverse taxa dramatically increase their feeding rate (both in absolute terms and relative to their body size) to meet their energetic needs during pregnancy and lactation (Shirley, 1984, Morgan and Winick, 1981, Fontaine, 2012). This is a strategy termed ‘income breeding’. Not all mammals follow this strategy though, with numerous mammal species in highly seasonal environments following a ‘capital breeding’ strategy whereby reserves are stored prior to reproduction (Stephens et al., 2009).

DEB theory suggests that the placenta increases a pregnant female’s surface area so that *f* = 1 corresponds to higher absolute food consumption (Kooijman, 2009). However, the food consumption of female rats peaks after the placenta has been expelled. During lactation, it reaches around 35 g × day ^−1^ (Shirley, 1984, Morgan and Winick, 1981), almost double the upper asymptote when the logistic function was fitted to observed feeding for females in this study. Without using feeding data, such patterns cannot possibly be simulated.

In Method 2, previously employed in Martin et al. (2019), *f* is a dynamic input calculated based on observed area specific feeding rate over time. However, as before, the values of $y_{\mathrm{AX}}$ and $J_{\mathrm{Xm}}^{a}$ remain fixed as animals grow. Using this method, $W_{R}$ relative to $W_{w}$ was substantially lower than body fat percentages reported in the literature (Reed et al., 2011, Rojas et al., 2018, Tekus et al., 2018) although observed patterns in fat storage over time were reflected by the model (Fig. 5). As previously noted, the observed negative relationship between body size and area specific feeding rate leads to *f* values that decline to well below one. Fitted parameter values must compensate for this and as a result, $V_{m}$ is extremely high. The parameter values in this study meant that, in theory, male rats could grow to almost 13 kg if they fed at a sufficiently high rate. This suggests that this method is fundamentally flawed, as such sizes are far beyond the highest observations in the literature (Hubert et al., 2000, Rojas et al., 2018).

Method 3 attempts to address issues with both the previous methods by positing that the values of $y_{\mathrm{AX}}$ and $J_{\mathrm{Xm}}^{a}$ vary as functions of body size and allowing *f* to fluctuate and exceed one. This attempt seems to have been largely successful. Using this method, modelled percentage body weight given by $W_{R}$ was closest to observed body fat percentage for both sexes at all but one timepoint. Moreover, the modelled maximum wet weight of structure, $W_{\mathrm{Vmw}}$, was only slightly lower than estimated peak lean weight in the calibration dataset.

### 4.4 Data limitations

Conventionally, DEB parameter estimations uses data for growth alongside reproduction data (Kooijman et al., 2008). This was not possible as our datasets did not include reproduction. However, for this study a combination of body weight and body length data would have been more informative, since our model assumes that the 1- *κ* fraction of assimilates is not specifically allocated to reproduction (an assumption that is consistent with observations (Morgan and Winick, 1981)). Length data would have allowed differentiation between weight gain due to structural growth or fat storage, allowing the division of total mass into reserve and structure. Unfortunately, length is not measured in standard dietary toxicity studies on rats (OECD, 1998, OECD, 2008, OECD, 2018b).

The lack of length measurements had two consequences. The first was that the parameter *κ* could not be estimated. In light of this, we used a value taken from the AmP library (Rakel and Gergs, 2018). It could be argued that this was not an appropriate choice, as differences in model equations and assumptions mean that *κ* performs a different function in the full DEB model. This issue is discussed in detail in the Supporting Information. However, we believe that this value at least provided a useful approximation in the absence of other options and was certainly preferable to the DEBkiss default - which would be more suitable for egg laying invertebrates with high reproductive investment. The over prediction of body fat percentage by Method 1 and to a lesser extent Method 3, suggest that, if anything, *κ* = 0.9472 was too low. Given that its maximum value is 1, there was little scope for any increase to affect results in a major way.

The second consequence of only having weight data was that our calculations of $J_{X}^{a}$ relied on the assumption that $a={\left(W_{w}/d_{w}\right)}^{2/3}$. Although the model could distinguish between reserve and structure, the same assumption was still necessary when calculating *f* in Methods 2 & 3 (so as to be consistent with calculations using data). While this was not ideal, this would have very little impact on results. In Method 2, $J_{\mathrm{Xm}}^{a}$ was more than double observed $J_{X}^{a}$ at larger body sizes (supporting fig. S38). Any discrepancy between $W_{w}/d_{w}$ and *V* would be relatively minor in comparison, so the resulting *f* values and overall decreasing trend would only be slightly affected. Using Method 3, the effect would be smaller still. Modelled $J_{X}$ (Fig. 2i & ii) begins to plateau at about 40 cm^2^ (equivalent to 253 g body weight) in male rats, and at about 30 cm^2^ (equivalent to 164 g body weight) in females. This means that from only a few weeks into the study period - when animals are assumed to have little reserve accumulated - the same level food of consumption (g_(*food*)_ × day^−1^) was expected regardless of body size. Therefore, correcting for differences between $W_{w}/d_{w}$ and *V* would not have any impact on the value of *f* for almost all of the observation period.

### 4.5 What issues remain?

Conventionally, digestive efficiency, $y_{\mathrm{AX}}$, is treated as a primary (fixed) parameter (Jager et al., 2013, Kooijman et al., 2008) but Method 3 changes this, such that this value increases with body size. This is highly plausible; several literature studies report increases in digestive efficiency associated with body size in a range of species (Smith, 1995, Illius and Gordon, 1992, Hansson and Jaarola, 1989, Demment and Vansoest, 1985). This occurs because increased gut capacity of larger animals allows the same volume of food to attain a greater surface area, while increased gut length leads to increased retention time for the extraction of nutrients.

In lieu of digestive efficiency data for rats, the generalised logistic model was fitted to food consumption data. This relationship relies on the assumption that growing rats, supplied with food *ad libitum*, consume enough food for area specific assimilation to equal $J_{\mathrm{Am}}^{a}$ and for structural volume to reach $V_{m}$. This appeared to be most true of male animals, with growth predictions being more accurate than for females. This would be consistent with the behavioural ecology of the species. Whereas females do not compete for mates and tend not to migrate, heavier males fare better in competition for dominance with unfamiliar individuals so there is selective pressure to grow as large as possible (Macdonald et al., 1999).

However, predictions with Method 3 were not always accurate for males either. Generally, predictions matched data well up until around day 500 but substantial deviations from data did occur thereafter. One possible explanation is that predicted $J_{\mathrm{Xm}}^{a}$ was too low at large body sizes making even small deviations from predictions proportionally larger than they should have been. This would exaggerate fluctuations in *f* and therefore ${∆W}_{w}$ in the later stages of growth. Another possibility though, is that this occurred because the model allowed structural growth in older animals despite skeletal growth in rats generally ceasing after around 6 months; a process that appears related to age rather than body size (Roach et al., 2003). This certainly contributed to higher assimilation and reserve accumulation late on.

This issue is particularly important when considering upregulated feeding during, or before, reproduction. In its current form, the model would permit structural growth when the additional assimilates should be allocated to foetal growth, milk production or increased energy storage in females or increased activity (maintenance costs) for intrasexual competition and defence of mates, in the case of males (Stephens et al., 2009). DEB theory actually suggests that the costs of foetal growth should be added to the mother’s maintenance costs rather than being paid from the 1-*κ* branch (Kooijman, 2009). For income breeders, such a model design might be enough to circumvent any problem since maintenance takes precedence over growth. However, a solution that would also apply to capital breeders would be to remove kappa from the model once growth has ceased. During growth, kappa plays the important role of determining the proportion of available assimilates that are stored or allocated to growth. When growth is complete however, allocation rules could be changed so that any assimilates that are not required for maintenance are simply be stored. The point at which this change to model rules is implemented would need to be based on species knowledge. Additionally, this would need to be implemented in such a way that regrowth after starvation could still occur.

A relatively minor issue is our lack of knowledge around weight loss and starvation in rats. Based on the data in this study and the literature, it appears to be typical for rats to reduce feeding and lose weight as body fat as they approach two years of age (Hubert et al., 2000, Tekus et al., 2018). This weight loss was overestimated by the model for males in group A. A possible reason is that reduced feeding elicits compensatory physiological or behavioural responses not included in the model’s starvation rules. For example reduced body temperature has been documented as a response to short-term starvation in rats (Sakurada et al., 2000), which would correspond to a reduction in maintenance rate, $J_{M}^{V}$. Finding the data needed to refine the starvation rules proposed by DEBkiss represents a challenge though. While some studies have restricted food availability (Hubert et al., 2000), enforcing longer term starvation leading to weight loss would be unethical due to the suffering this would cause.

In order to address the remaining issues, the clear solution is to simply measure digestive efficiency of standard laboratory diets (Batzli and Cole, 1979, Veloso and Bozinovic, 1993) alongside food consumption and body weight in growing rats. This would allow the relationship between $J_{\mathrm{Xm}}^{a}$ and body size to be determined mechanistically and for Eq. 11 to be solved, providing the value of the maximum assimilation rate $J_{\mathrm{Am}}^{a}$. Inevitably, empirical relationships can only provide an imperfect representation of reality. Indeed, at extreme body sizes (>1.577 kg for males and >1.567 kg for females) our parameters mean that $J_{\mathrm{Xm}}^{a}<J_{\mathrm{Am}}^{a}$ and therefore $y_{\mathrm{AX}}>1$. This is a physical impossibility as assimilates from food cannot exceed the mass of the food itself. The strong performance of Method 2 in predicting independent data suggests that the reality may sit between Methods 2 and 3. It appears likely that $J_{\mathrm{Xm}}^{a}$ does decrease as animals grow, though less dramatically than Method 3 predicts. Likewise, *f* likely falls as animals grow, but less markedly than suggested by Method 2.

## 5 Conclusions

DEBkiss (Jager et al., 2013) inevitably made some compromises in order to simplify the DEB framework. However, our results suggest that it is the calculation used to derive feeding inputs in all versions of DEB (Kooijman et al., 2008), which represents an over-simplification. This was designed to circumvent the need for detailed feeding data, which are rarely available (Kooijman, 2009, Van Der Meer, 2006). However, observed patterns between the feeding rate and surface area of rats clearly contradict model assumptions and so changes are required.

We have developed methods which extract more information from feeding data in order to broaden the applicability of models based on DEBkiss. With this approach we have produced accurate and biologically sound models that use simple equations to model growth and reserve dynamics. This removes the assumption of first order dynamics of reserve density, which is the most difficult aspect of the full DEB growth model (Van der Meer, 2006). Where feeding data are unavailable, conventional methods by which constant or simple *f* inputs are assumed, may still be most suitable. However, we suggest that $y_{\mathrm{AX}}$ and $J_{\mathrm{Xm}}^{a}$ are dynamic variables that vary with surface area and that, even if these relationships cannot be quantified for most species, DEB theory should reflect this.

While the new method is a significant step in the right direction, relying on empirical relationships is not ideal and several issues remain that could be addressed by data collection. Models able to accurately predict how animals in dietary toxicity studies would have grown if fed a control diet are now within reach. Such models are a prerequisite for DEB-TKTD models that accurately reflect a compound’s toxicity. Equally though, they represent an exciting new tool with which to analyse toxicological data, avoiding the conflation of effects due to toxicity and differences in feeding rate. This will allow assessment of how feeding avoidance impacts upon the ecological risk posed by a chemical in a way that was not previously possible.

## Funding Information

This project is supported by BBSRC Industrial Case Studentship BB/P504944/1 in partnership with Syngenta and hosted by The University of York.

## CRediT authorship contribution statement

**Thomas Martin:** Conceptualization, Data curation, Methodology, Software, Writing – original draft. **Mark E Hodson:** Conceptualization, Writing – review & editing, Supervision. **Roman Ashauer:** Conceptualization, Writing – review & editing, Supervision, Methodology.

## Declaration of Competing Interest

The authors declare that they have no known competing financial interests or personal relationships that could have appeared to influence the work reported in this paper.
