# Supplementary material for: Modelling the effects of variability in feeding rate on growth – a vital step for DEB-TKTD modelling
Source: Ecotoxicol Environ Saf. 2022 Mar 1;232:113231. doi: 10.1016/j.ecoenv.2022.113231 (PMC8873987; doi:10.1016/j.ecoenv.2022.113231)
Supplement: Supplementary file 1 — Supplementary material. [file mmc1.docx]

**Supporting Information for: Modelling effects of variability in feeding rate on growth – a vital step for DEB-TKTD modelling**

Thomas Martin^1🖂^ , Mark E Hodson^1^, Roman Ashauer^1,2^

1. University of York, Environment Department, Heslington, York, YO10 5NG, UK
2. Syngenta Crop Protection AG, Basel, 4002, Switzerland

🖂 [tomartin604@gmail.com](mailto:tomartin604@gmail.com)

**Contents**

Real time *f* calculation worked example Page S3

The role of kappa in the DEBkiss and full DEB models Page S5

**Supporting Tables** Page S7

**Supporting Figures** Page S8

Figures S1 - S6: Modelled Wet Weight – Calibration Data Page S10

Figures S7 - S18: Predicted Wet Weight – Calibration Data Page S11

Figures S19 - S24: Modelled Structure & Reserve – Calibration Data Page S17

Figures S25 - S36: Predicted Structure & Reserve – Independent Data Page S20

Figures S37 -S38: Additional Feeding Data Plots Page S26

**Real time *f* calculation: worked example**

For Methods 2 and 3, the scaled feeding rate *f* was calculated in real time as the growth model ran. This was not applicable to Method 1 as *f* was not derived from feeding data in that approach. This was in order to ensure that the models reflected growth based on the quantity of food consumed, rather than observed area specific feeding rate which could lead to a ‘snowball effect’ where predictions deviate from data (figure 1). Hypothetical data were used to illustrate the impact this had on modelled $\Delta W_{w}$ (using the fitted parameter values given in table 1 of the main paper). A male rat was considered, weighing 300g and consuming 24g food per day, with Methods 2 and 3 returning *f* values 0.65 and 0.98 respectively (table S1).

**Table S1** Calculations of scaled feeding rate, *f*, using Methods 2 and 3 for the same hypothetical data.

| Method | $J_{X}$,  g*_food_* × day^-1^ | $W_{w}$,  g | $J_{X}^{a}$,  g*_food_* × cm^-2^ × day^-1^ | $J_{Xm}^{a}$  (Method 2),  g*_food_* × cm^-2^ × day^-1^ | Predicted $J_{Xm}^{a}$ at 300g  (Method 3), g*_food_* × cm^-2^ × day^-1^ | *f* |
| --- | --- | --- | --- | --- | --- | --- |
|  |  |  |  |  |  |  |
| 2 | 24 | 300 | 0.54 | 0.82 | N/A | 0.65 |
|  |  |  |  |  |  |  |
| 3 | 24 | 300 | 0.54 | N/A | 0.55 | 0.98 |
|  |  |  |  |  |  |  |

We then supposed that modelled $W_{w}$ was either accurate or 50g above or below that observed at time *t* (in all cases $W_{w}$ was broken down into 88% structure and 12% reserve) and calculated $\Delta W_{w}$ for the next time step (table S2). Using Method 2 and the *f* value derived directly from data, as in Martin *et al.* (2019), showed that *f* always corresponds to the same area specific feeding rate, $J_{X}^{a}$, equating to a higher $J_{X}$ in larger animals and vice versa. This leads to positive feedback between modelled $W_{w}$ and $\Delta W_{w}$. Therefore, once predictions deviate from data, they become less accurate with each time step until *f* is changed. Using Method 3, the value of $J_{X}^{a}$ corresponding to the given value of *f* decreases with body size. In this case, negative feedback occurs between modelled $W_{w}$ and $\Delta W_{w}$ at a given value of *f*. However, a positive relationship exists between modelled $W_{w}$ and $J_{X}$, so $\Delta W_{w}$ does not exactly reflect the quantity of food consumed.

**Table S2** Food consumption, $J_{X}$, and growth rate, $\Delta W_{w}$, as calculated from predicted body weight, $W_{w}$, and observed scaled feeding rate, *f*, using Methods 2 and 3. This approach was not used in this study due to positive feedback.

| Method | Inputted *f* value | Predicted $W_{w}$ at time *t,*  G | Corresponding $J_{X}^{a}$ at time *t*,  g_(_*_food_*_)_ × cm^-2^ × day^-1^ | Corresponding $J_{X}$ at time *t*,  g_(_*_food_*_)_ × day^-1^ | Predicted $\Delta W_{w}$,  g × day^-1^ |
| --- | --- | --- | --- | --- | --- |
| Method 2 | 0.65 | 350 | 0.54 | 26.60 | 1.68 |
|  | 0.65 | 300 | 0.54 | 24.00 | 1.57 |
|  | 0.65 | 250 | 0.54 | 21.25 | 1.44 |
| Method 3 | 0.98 | 350 | 0.49 | 24.48 | 1.60 |
|  | 0.98 | 300 | 0.54 | 24.00 | 1.68 |
|  | 0.98 | 250 | 0.59 | 23.27 | 1.72 |

This exercise was then repeated, but *f* was instead derived from observed food consumption, $J_{X}$, and modelled $W_{w}$ (table S3). This was the approach used throughout this study. When the calculations are performed in this order, $J_{X}^{a}$ and *f* decrease as modelled body size increases, and vice versa. For method 2, this curtails the ‘snowball effect’, instead leading to negative feedback between modelled $W_{w}$ and $\Delta W_{w}$ at a given value of $J_{X}$. For method 3, negative feedback occurs between $W_{w}$ and $\Delta W_{w}$ is strengthened (a difference of 0.44 g × day^-1^ between $\Delta W_{w}$ at 250g and 350g body weight) as predicted growth rate accurately reflects observed daily food consumption.

**Table S3** Scaled feeding rate, *f*, and growth rate, $\Delta W_{w}$, calculated from predicted body weight, $W_{w}$, and observed food consumption, $J_{X}$, using Methods 2 and 3. This is the approach used in this study.

| Method | Observed $J_{X}$ at time *t,*  g_(_*_food_*_)_ × day^-1^ | Predicted $W_{w}$ at time *t,*  (g) | Corresponding $J_{X}^{a}$  at time *t*,  g_(_*_food_*_)_ × cm^-2^ × day^-1^ | Inputted *f* value | Predicted $\Delta W_{w}$,  g × day^-1^ |
| --- | --- | --- | --- | --- | --- |
|  |  |  |  |  |  |
| 2 | 24 | 350 | 0.48 | 0.59 | 1.40 |
|  | 24 | 300 | 0.54 | 0.65 | 1.57 |
|  | 24 | 250 | 0.60 | 0.74 | 1.74 |
| 3 | 24 | 350 | 0.48 | 0.96 | 1.46 |
|  | 24 | 300 | 0.54 | 0.98 | 1.68 |
|  | 24 | 250 | 0.60 | 1.01 | 1.90 |

**The role of kappa in the DEBkiss and full DEB models**

The full DEB growth model (Van der Meer, 2006) can be represented as

$$\begin{aligned} \frac{dV}{dt}=\frac{k\dot{P}_{C}-{\{\dot{P}}_{T}\}V^{\frac{2}{3}}-{[\dot{P}}_{M}]V}{\left[ E_{G} \right]}\#1 \end{aligned}$$

Where $\dot{P}_{C}$ is the utilisation rate from the reserves (J × t^-1^), ${\{\dot{P}}_{T}\}$ is maintenance rate per unit area (J × cm^-2^× t^-1^), ${[\dot{P}}_{M}]$ is maintenance rate per unit volume (J × cm^-3^ × t^-1^), and $\left[ E_{G} \right]$ is the energetic cost per unit growth (J × cm^-2^).

Like the AmP entry, we assumed that the experiments took place within the thermoneutral zone for the rats and so area specific maintenance (heating) costs were zero. Therefore, the model can be represented as

$$\begin{aligned} \frac{dV}{dt}=\frac{1}{\left[ E_{G} \right]}\left( k\dot{P}_{C}-{[\dot{P}}_{M}]V \right)\#2 \end{aligned}$$

In DEBkiss (Jager *et al.*, 2013), the assimilation flux, $J_{A}$ is given by $fJ_{Am}^{a}V^{2/3}$ where $J_{Am}^{a}$ is the maximum surface area specific assimilation rate (g_(assimilates)_ × cm_(_*_L_*_)_^-2^ × d^-1^), *V* is volume (cm^3)^ and *f* is scaled feeding rate. Substituting this into the growth model gives the equation

$$\begin{aligned} \frac{dV}{dt}=y_{VA}\left( kJ_{A}-J_{M}^{V}V \right)/d_{V}\#3 \end{aligned}$$

where $J_{M}^{V}$ is the mass specific maintenance rate (g_(assimilates)_ × cm_(_*_L_*_)_^-3^ × d^-1^), $y_{VA}$ is the yield of structure over assimilates or growth efficiency (g_(structure)_ × g_(assimilates)_^-1^) and $d_{V}$ is density of structure (g × cm^-3^). This is very similar to the full DEB model given in equation 2. Both can be summarised as

$$\begin{aligned} Growth Rate= Growth Efficiency \times\left( Supply to Soma - Maintenance Flux \right)\#4 \end{aligned}$$

**Kappa**

As to whether *κ* performs the same function in growth model, the key question is whether $J_{A}$ in DEBkiss can be considered analogous to $\dot{P}_{C}$ in the full DEB model. In the sense that both represent the total flux of mobilised resources (whether reserve or assimilates) these parameters do describe the same thing. Therefore, in both models *κ* describes the fraction of available resources allocated to somatic maintenance and growth while (1- *κ*) represents the fraction allocated to other processes.

However, the list of ‘other’ processes covered by (1- *κ*) does differ between the two models. In the full DEB model, this fraction covers reproduction and maturation, while in our DEBkiss based model it covers storage. This is because, in the full DEB model reserve storage occurs immediately after assimilation and before reserve mobilisation and *κ* partitioning. In this sense, $\dot{P}_{C}$ and $J_{A}$ represent different processes and so *κ* is performing a different function in each model. Therefore, a closer examination of whether the AmP value of *κ* was appropriate is warranted.

Generally, data for body size (weight or length) and reproduction are used to estimate the relative investment into somatic maintenance and growth vs reproduction, and so derive *κ*. The reproductive investment of female placental mammals occurs primarily during pregnancy and lactation, with energetic costs being met through an increase or change in feeding (Bernard & Hohn, 1989; Fontaine, 2012) rather than through utilising a reproduction buffer amassed through *κ* partitioning. The feeding of female Sprague-Dawley rats (Shirley, 1984; Morgan & Winick, 1980) has been shown to increase dramatically during pregnancy and lactation, up to a peak of around 35g/day (around double the upper asymptote of the generalised logistic curve when fitted to feeding data for unfertilised female rats in our calibration dataset).

Considering this, and how our growth model was structured, body size and reproduction data would not be the combination best suited to estimate *κ*. Instead, body weight alongside length or body fat percentage would be better as this would allow differentiation between structural growth and weight gain due to reserve storage. Unfortunately, neither length nor fat percentage are routinely measured in any OECD test protocols (OECD, 2001, 2018a, 2018b).

The calibration dataset in our study did not include reproduction either so the only option, other than using the AmP value, would be to fix *κ* at its default of 0.8 which would not have been suitable for several reasons. DEBkiss was developed with invertebrates in mind, with the (1- *κ*) fraction of assimilates stored as mass for egg production in the reproduction buffer. Placental mammals do not need mass for eggs and so higher value of *κ* would be expected.

The value, *κ* = 0.9472, given by the AmP entry suggests high investment into the soma and only a small fraction of assimilates stored for future reproductive investment, which would be more consistent with mammalian reproduction. Moreover, independent observations of body fat percentage over time were closely matched by the predictions of Method 3 (the only method which was responsive to feeding variability and indicated a realistic maximum weight), suggesting that this value of *κ* was reasonably accurate. Finally, the fact that reported body fat percentages were similar for male and female rats would suggest that using the same *κ* value for males and females in our model was also justified.

**References**

BERNARD, H. J. & HOHN, A. A. 1989. Differences in Feeding Habits between Pregnant and Lactating Spotted Dolphins (Stenella attenuata). *Journal of Mammalogy,* 70, 211-215.

FONTAINE, E. 2012. Food Intake and Nutrition During Pregnancy, Lactation and Weaning in the Dam and Offspring. *Reproduction in Domestic Animals,* 47**,** 326-330.

JAGER, T., MARTIN, B. T. & ZIMMER, E. I. 2013. DEBkiss or the quest for the simplest generic model of animal life history. *Journal of Theoretical Biology,* 328**,** 9-18.

KOOIJMAN, S., SOUSA, T., PECQUERIE, L., VAN DER MEER, J. & JAGER, T. 2008. From food-dependent statistics to metabolic parameters, a practical guide to the use of dynamic energy budget theory. *Biological Reviews,* 83**,** 533-552.

MORGAN, B. & WINICK, M. 1981. A possible control of food intake during pregnancy in the rat. *British Journal of Nutrition,* 46**,** 29-37.

OECD 2001. Test No. 416: Two-Generation Reproduction Toxicity.

OECD 2018a. Test No. 451: Carcinogenicity Studies.

OECD 2018b. Test No. 452: Chronic Toxicity Studies

SHIRLEY, B. 1984. The food intake of rats during pregnancy and lactation. *Laboratory Animal Science,* 34**,** 169-172.

VAN DER MEER, J. 2006. An introduction to Dynamic Energy Budget (DEB) models with special emphasis on parameter estimation. *Journal of Sea Research,* 56**,** 85-102

**Supporting Tables**

**Table S4** Summary data based on mean observed body weight and food consumption at each timepoint.

| Sex | Male | | | Female | | |
| --- | --- | --- | --- | --- | --- | --- |
| Dataset | A  (Calibration) | B | C | A  (Calibration) | B | C |
| Total Food Consumption (kg) | 17.97 | 18.36 | 17.32 | 12.24 | 13.76 | 12.16 |
| Initial Weight (g) | 113.37 | 209.38 | 190.35 | 104.14 | 189.22 | 144.69 |
| Max Weight (g) | 839.04 | 859.78 | 695.08 | 486.21 | 559.79 | 433.77 |
| Final Weight (g) | 742.35 | 784.36 | 672.11 | 464.31 | 549.12 | 430.20 |
| Weight gain (g) | 628.99 | 574.98 | 481.76 | 360.17 | 359.90 | 285.51 |

**Table S5** A comparison of maximum wet weight of structure, $W_{Vmw}$, estimated from data and calculated from model parameters.

| Sex | Male | | | Female | | |
| --- | --- | --- | --- | --- | --- | --- |
| Estimated $W_{Vmw}$ (g) | 656.97 | | | 380.7 | | |
| Method | 1 | 2 | 3 | 1 | 2 | 3 |
| Modelled $W_{Vmw}$ (g) | 481.5 | 12910 | 579.1 | 278.3 | 5422 | 356.5 |
| Model/ Estimate | 0.733 | 19.53 | 0.881 | 0.733 | 14.1 | 0.932 |

**Supporting Figures**

**Modelled Wet Weight – Calibration Data**

**Method 1 – Dataset A**

**Fig. S1** Wet weight of male rats in group A over two years, modelled using Method 1 (solid line), fitted to mean observations (black circles). Grey circles represent raw data while dashed lines show the observed mean ±1 SD.

**Fig. S2** Wet weight of female rats in group A over two years, modelled using Method 1 (solid line), fitted to mean observations (black circles). Grey circles represent raw data while dashed lines show the observed mean ±1 SD.

**Method 2 - Dataset A**

**Fig. S3** Wet weight of male rats in group A over two years, modelled using Method 2 (solid line), fitted to mean observations (black circles). Grey circles represent raw data while dashed lines show the observed mean ±1 SD.

**Fig. S4** Wet weight of female rats in group A over two years, modelled using Method 2 (solid line), fitted to mean observations (black circles). Grey circles represent raw data while dashed lines show the observed mean ±1 SD.

**Method 3 – Dataset A**

**Fig. S5** Wet weight of male rats in group A over two years, modelled using Method 3 (solid line), fitted to mean observations (black circles). Grey circles represent raw data while dashed lines show the observed mean ±1 SD.

**Fig. S6** Wet weight of female rats in group A over two years, modelled using Method 3 (solid line), fitted to mean observations (black circles). Grey circles represent raw data while dashed lines show the observed mean ±1 SD.

**Predicted Wet Weight – Independent Data**

**Method 1 – Dataset B**

**Fig. S7** Observed (circles) and predicted (solid line) wet weight of male rats in group B over two years, using Method 1. Mean observations are shown in black while grey circles represent raw data. Dashed lines show the observed mean ±1 SD.

**Fig. S8** Observed (circles) and predicted (solid line) wet weight of female rats in group B over two years, using Method 1. Mean observations are shown in black while grey circles represent raw data. Dashed lines show the observed mean ±1 SD.

**Method 1 – Dataset C**

**Fig. S9** Observed (circles) and predicted (solid line) wet weight of male rats in group C over two years, using Method 1. Mean observations are shown in black while grey circles represent raw data. Dashed lines show the observed mean ±1 SD.

**Fig. S10** Observed (circles) and predicted (solid line) wet weight of female rats in group C over two years, using Method 1. Mean observations are shown in black while grey circles represent raw data. Dashed lines show the observed mean ±1 SD.

**Method 2 - Dataset B**

**Fig. S11** Observed (circles) and predicted (solid line) wet weight of male rats in group B over two years, using Method 2. Mean observations are shown in black while grey circles represent raw data. Dashed lines show the observed mean ±1 SD.

**Fig. S12** Observed (circles) and predicted (solid line) wet weight of female rats in group B over two years, using Method 2. Mean observations are shown in black while grey circles represent raw data. Dashed lines show the observed mean ±1 SD.

**Method 2 - Dataset C**

**Fig. S13** Observed (circles) and predicted (solid line) wet weight of male rats in group C over two years, using Method 2. Mean observations are shown in black while grey circles represent raw data. Dashed lines show the observed mean ±1 SD.

**Fig. S14** Observed (circles) and predicted (solid line) wet weight of female rats in group C over two years, using Method 2. Mean observations are shown in black while grey circles represent raw data. Dashed lines show the observed mean ±1 SD.

**Method 3 – Dataset C**

**Fig. S15** Observed (circles) and predicted (solid line) wet weight of male rats in group B over two years, using Method 3. Mean observations are shown in black while grey circles represent raw data. Dashed lines show the observed mean ±1 SD.

**Fig. S16** Observed (circles) and predicted (solid line) wet weight of female rats in group B over two years, using Method 3. Mean observations are shown in black while grey circles represent raw data. Dashed lines show the observed mean ±1 SD.

**Method 3 – Dataset C**

**Fig. S17** Observed (circles) and predicted (solid line) wet weight of male rats in group C over two years, using Method 3. Mean observations are shown in black while grey circles represent raw data. Dashed lines show the observed mean ±1 SD.

**Fig. S18** Observed (circles) and predicted (solid line) wet weight of female rats in group C over two years, using Method 3. Mean observations are shown in black while grey circles represent raw data. Dashed lines show the observed mean ±1 SD.

**Modelled Structure, Reserve and Wet Weight– Calibration Data**

**Method 1 – Dataset A**


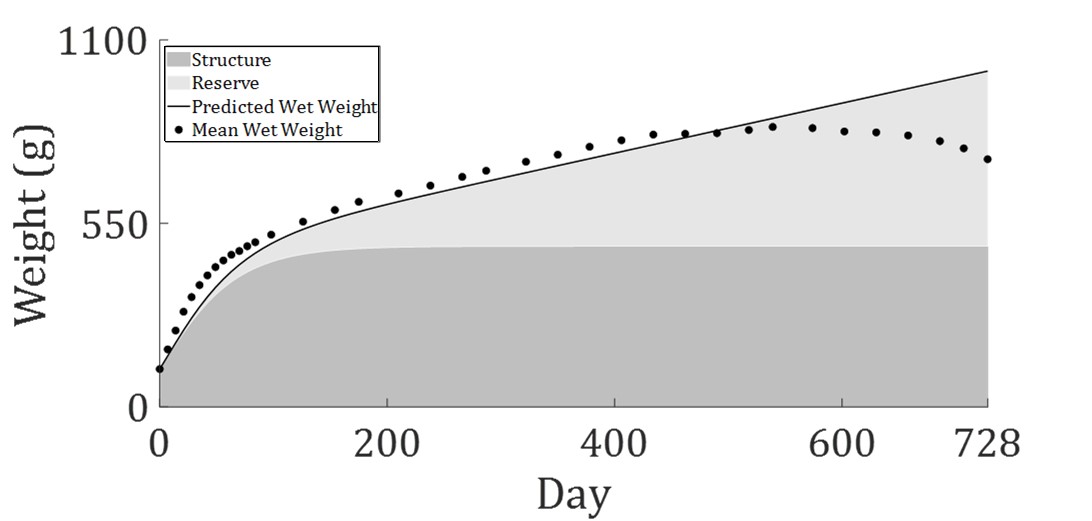


**Fig. S19** Wet weight of male rats in group A over two years, modelled using Method 1 (solid line), fitted to mean observations (black circles). The shaded area under the model curves shows structure (dark grey) and reserve (light grey).


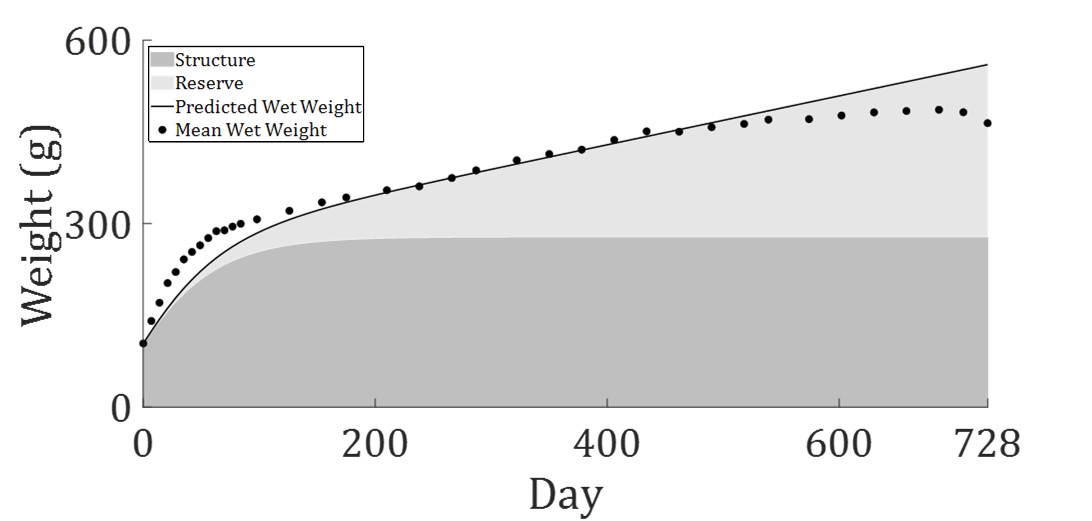


**Fig. S20** Wet weight of female rats in group A over two years, modelled using Method 1 (solid line), fitted to mean observations (black circles). The shaded area under the model curves shows structure (dark grey) and reserve (light grey).

**Method 2- Dataset A**


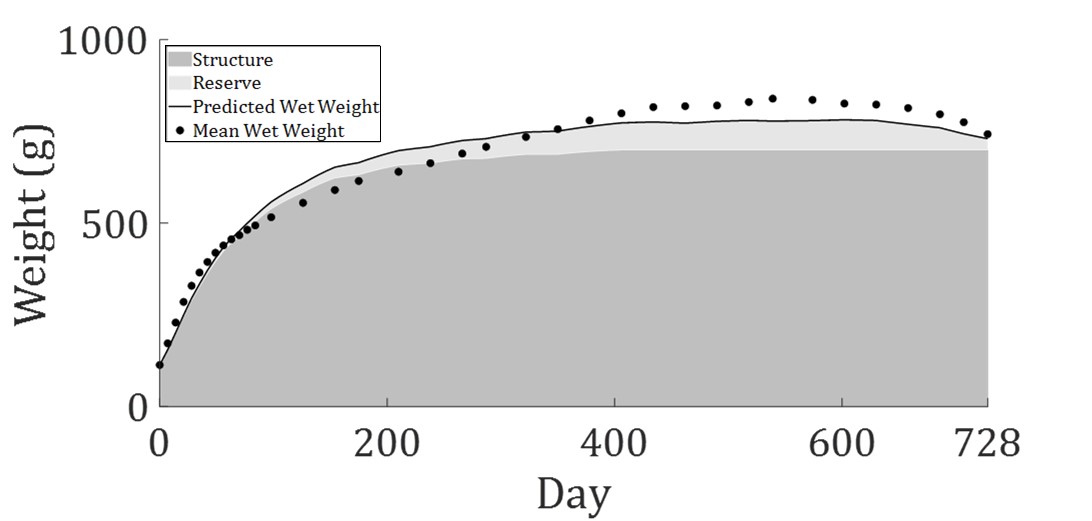


**Fig. S21** Wet weight of male rats in group A over two years, modelled using Method 2 (solid line), fitted to mean observations (black circles). The shaded area under the model curves shows structure (dark grey) and reserve (light grey).


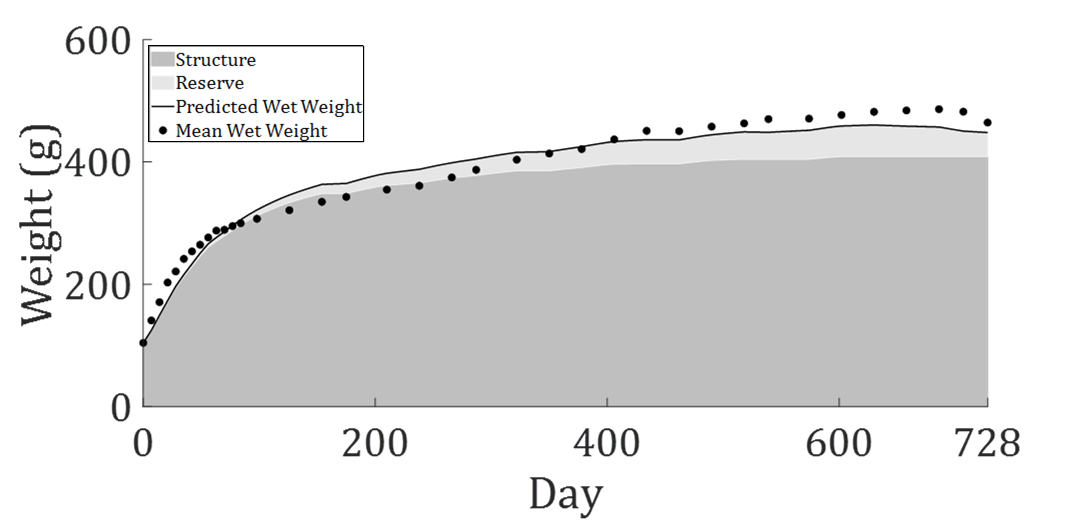


**Fig. S22** Wet weight of female rats in group A over two years, modelled using Method 2 (solid line), fitted to mean observations (black circles). The shaded area under the model curves shows structure (dark grey) and reserve (light grey).

**Method 3 – Dataset A**


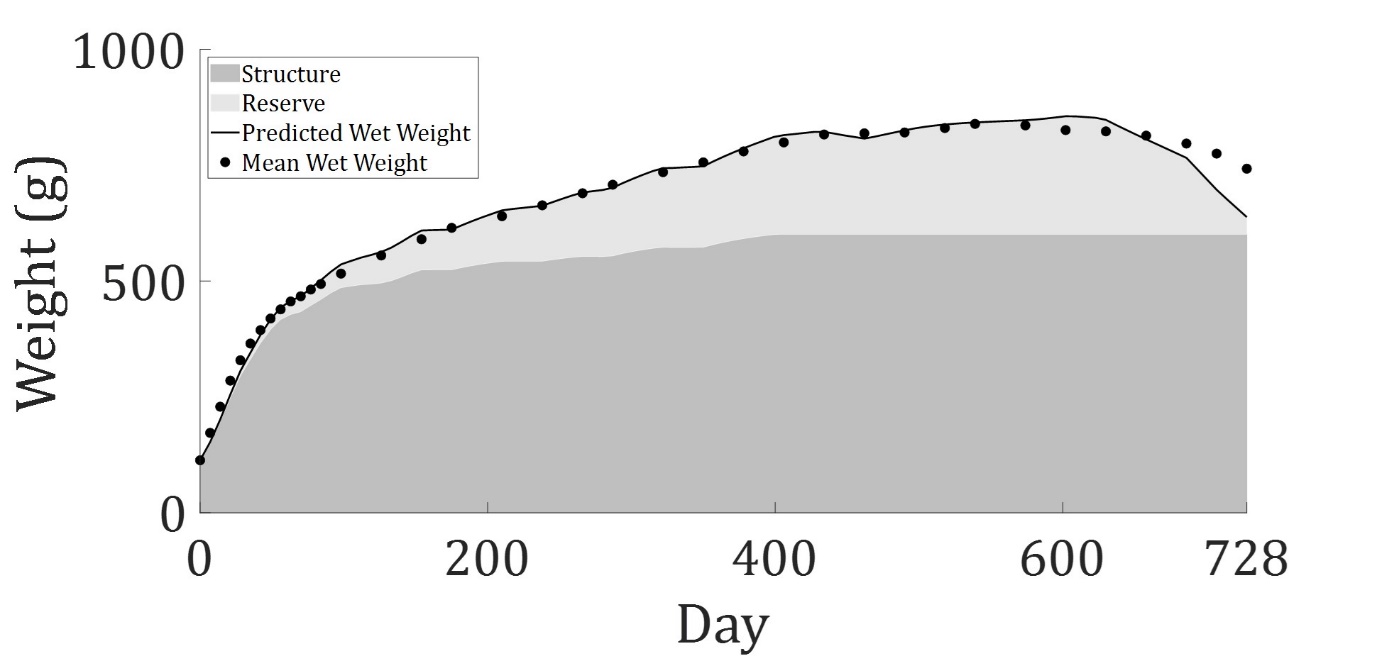


**Fig. S23** Wet weight of male rats in group A over two years, modelled using Method 3 (solid line), fitted to mean observations (black circles). The shaded area under the model curves shows structure (dark grey) and reserve (light grey).


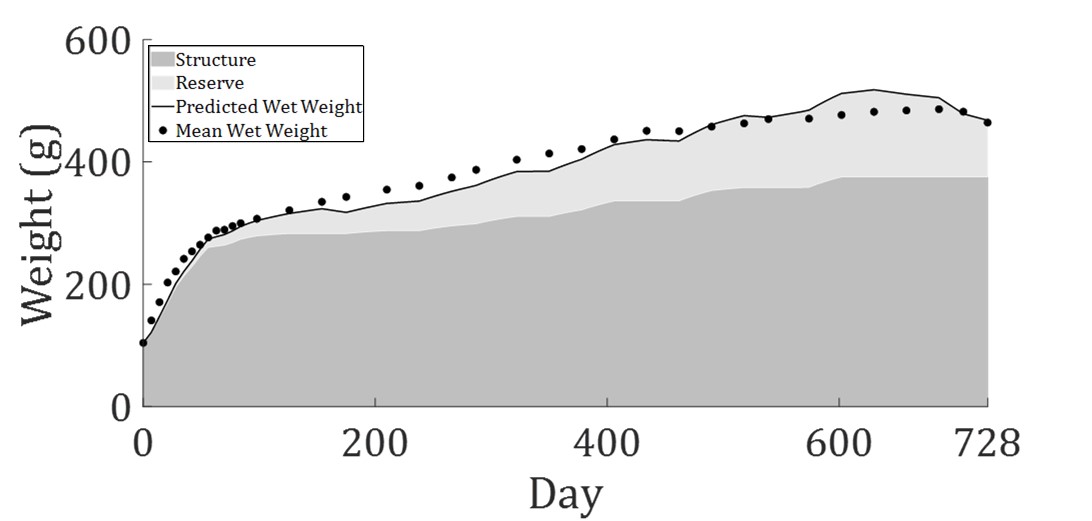


**Fig. S24** Wet weight of female rats in group A over two years, modelled using Method 3 (solid line), fitted to mean observations (black circles). The shaded area under the model curves shows structure (dark grey) and reserve (light grey).

**Predicted Structure, Reserve and Wet Weight – Independent Data**

**Method 1 – Dataset B**


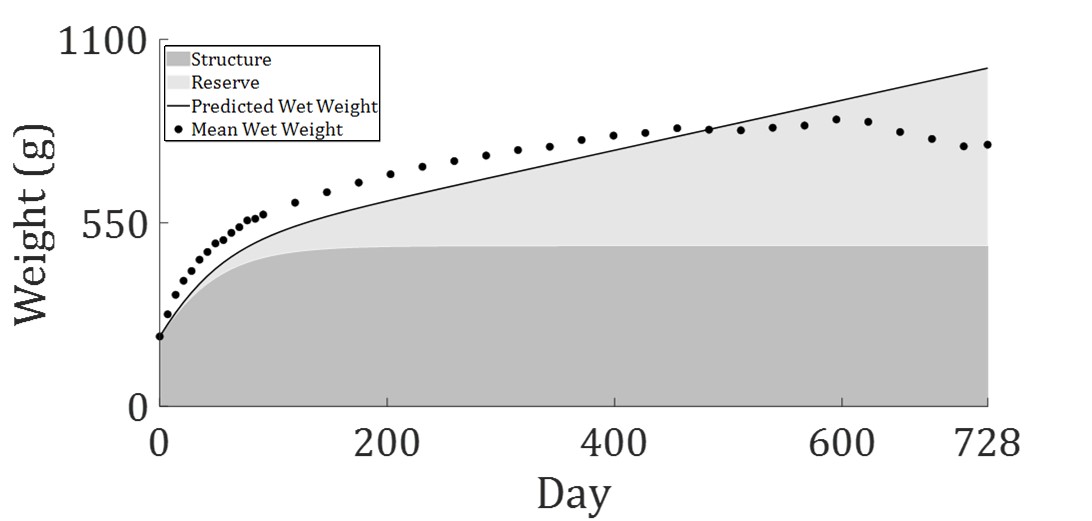


**Fig. S25** Observed (circles) and predicted (solid line) wet weight of male rats in group B over two years, using Method 1. The shaded area under the model curves shows structure (dark grey) and reserve (light grey).


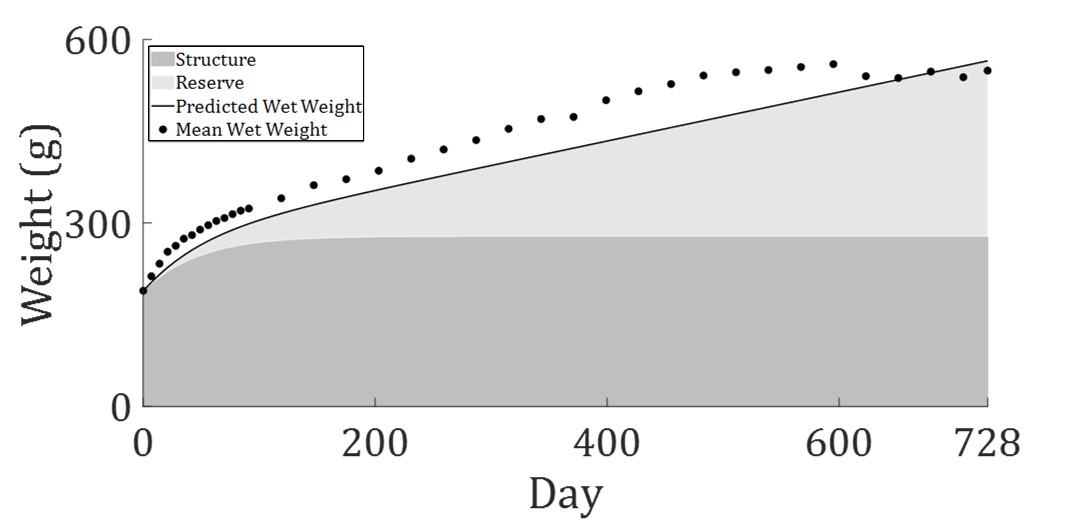


**Fig. S26** Observed (circles) and predicted (solid line) wet weight of female rats in group B over two years, using Method 1. The shaded area under the model curves shows structure (dark grey) and reserve (light grey).

**Method 1 – Dataset C**


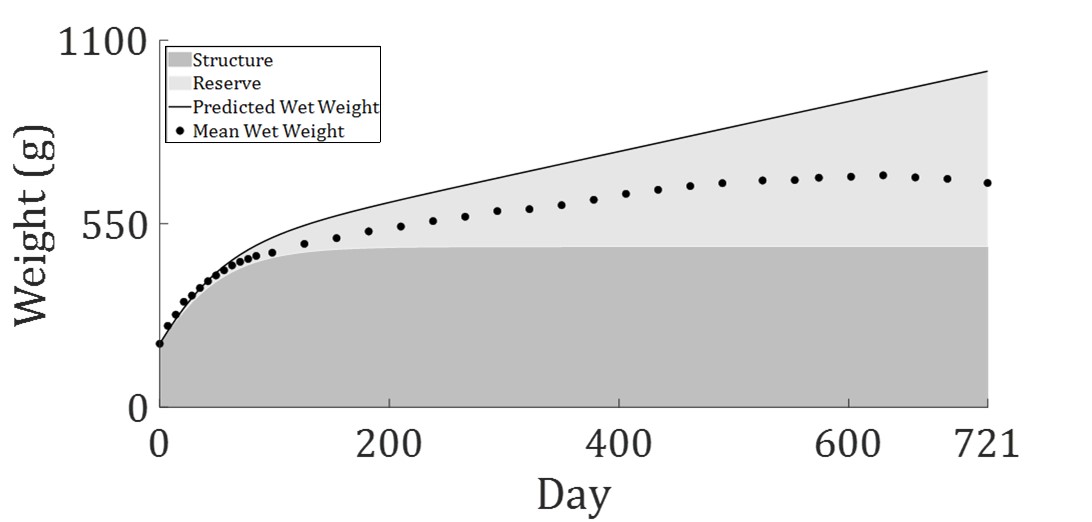


**Fig. S27** Observed (circles) and predicted (solid line) wet weight of male rats in group C over two years, using Method 1. The shaded area under the model curves shows structure (dark grey) and reserve (light grey).


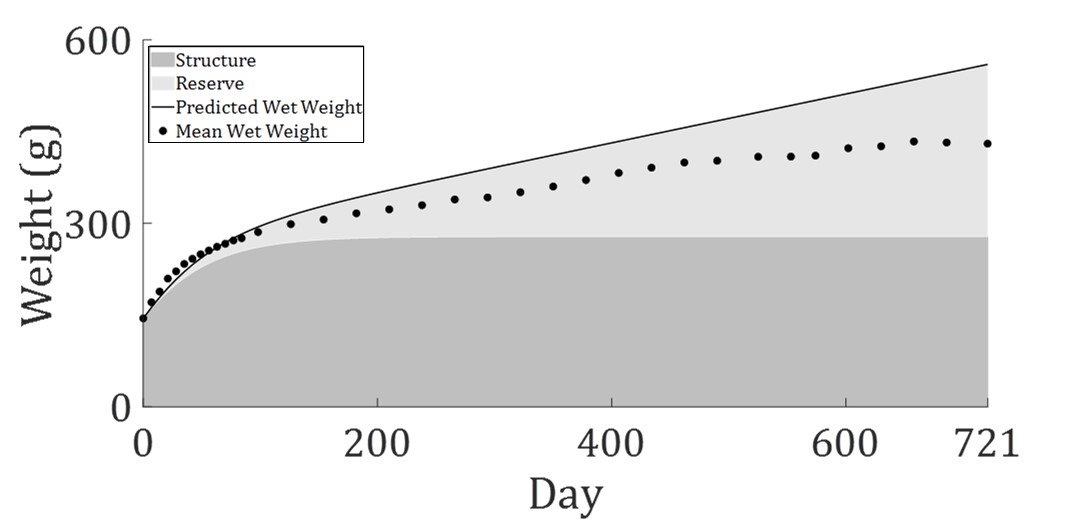


**Fig. S28** Observed (circles) and predicted (solid line) wet weight of female rats in group C over two years, using Method 1. The shaded area under the model curves shows structure (dark grey) and reserve (light grey).

**Method 2 - Dataset B**


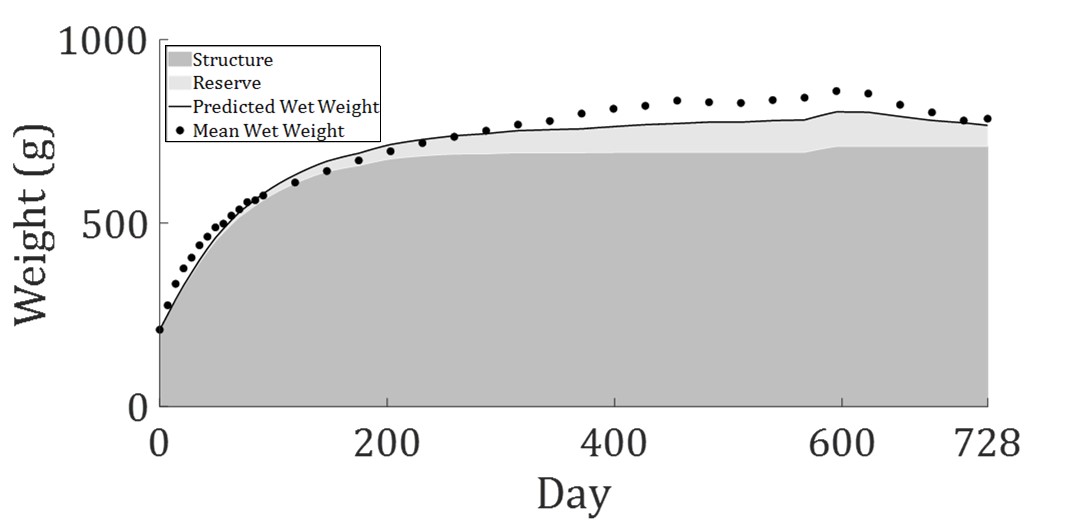


**Fig. S29** Observed (circles) and predicted (solid line) wet weight of male rats in group B over two years, using Method 2. The shaded area under the model curves shows structure (dark grey) and reserve (light grey).


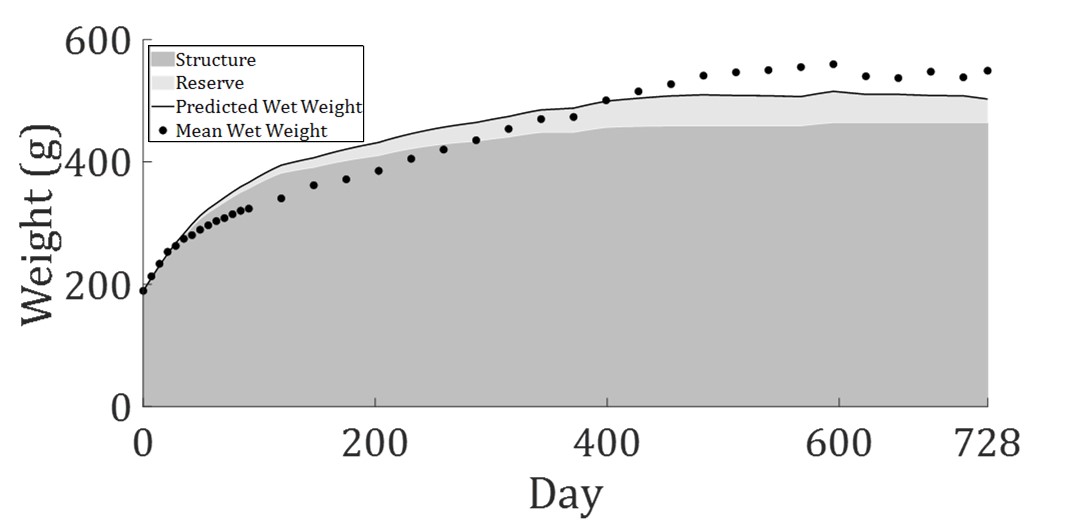


**Fig. S30** Observed (circles) and predicted (solid line) wet weight of female rats in group B over two years, using Method 2. The shaded area under the model curves shows structure (dark grey) and reserve (light grey).

**Method 2 - Dataset C**


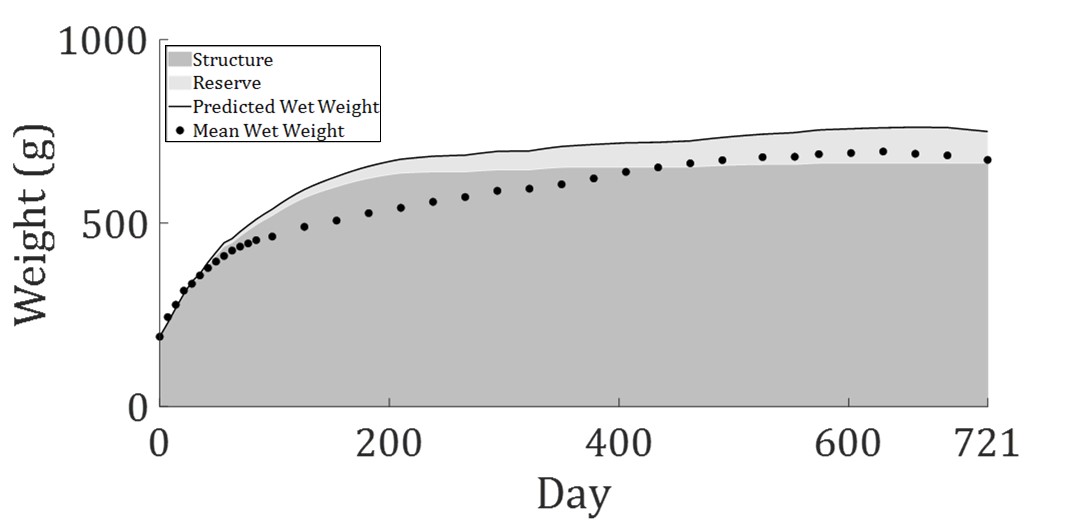


**Fig. S31** Observed (circles) and predicted (solid line) wet weight of male rats in group C over two years, using Method 2. The shaded area under the model curves shows structure (dark grey) and reserve (light grey).


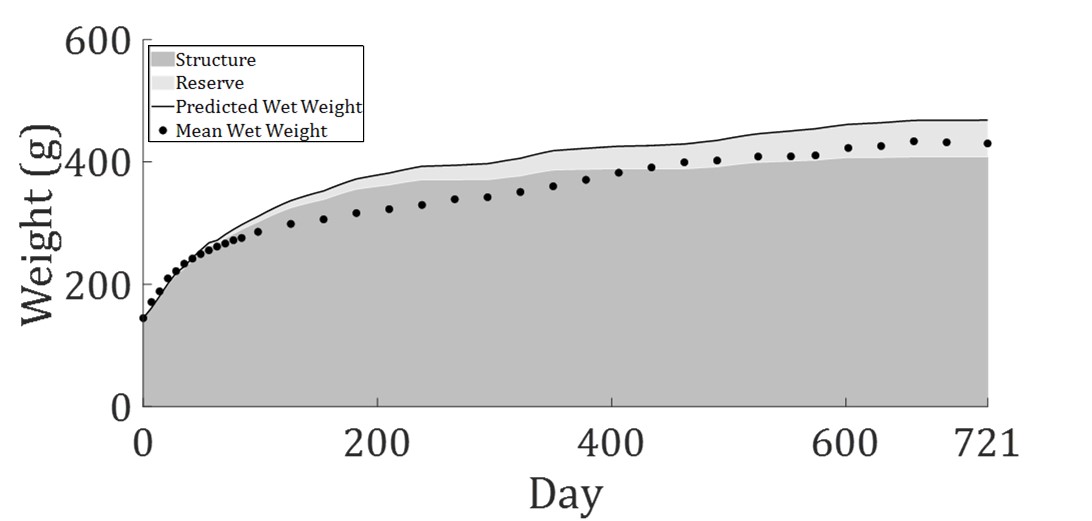
 **Fig. S32** Observed (circles) and predicted (solid line) wet weight of female rats in group C over two years, using Method 2. The shaded area under the model curves shows structure (dark grey) and reserve (light grey).

**Method 3 – Dataset C**


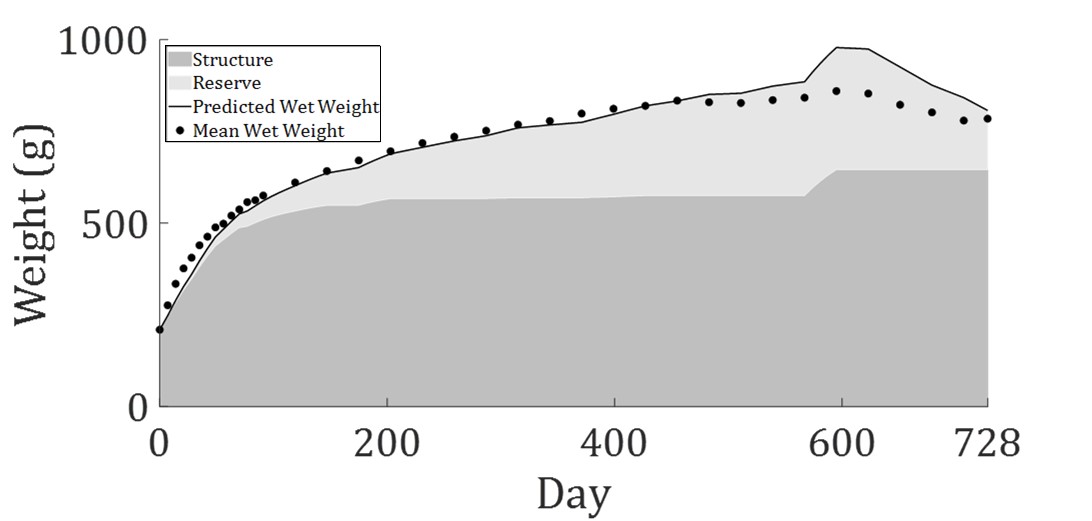


**Fig. S33** Observed (circles) and predicted (solid line) wet weight of male rats in group B over two years, using Method 3. The shaded area under the model curves shows structure (dark grey) and reserve (light grey).


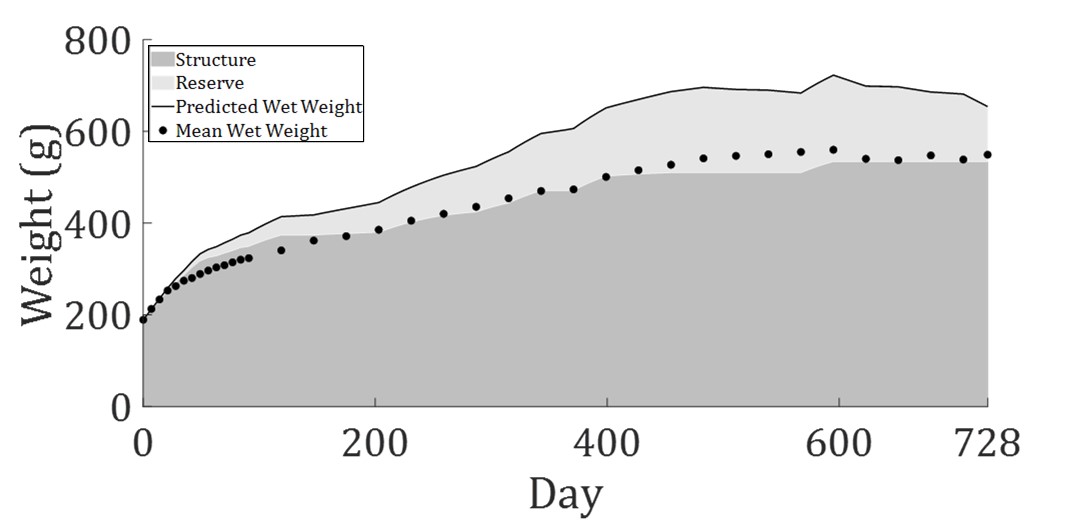


**Fig. S34** Observed (circles) and predicted (solid line) wet weight of female rats in group B over two years, using Method 3. The shaded area under the model curves shows structure (dark grey) and reserve (light grey).

**Method 3 – Dataset C**


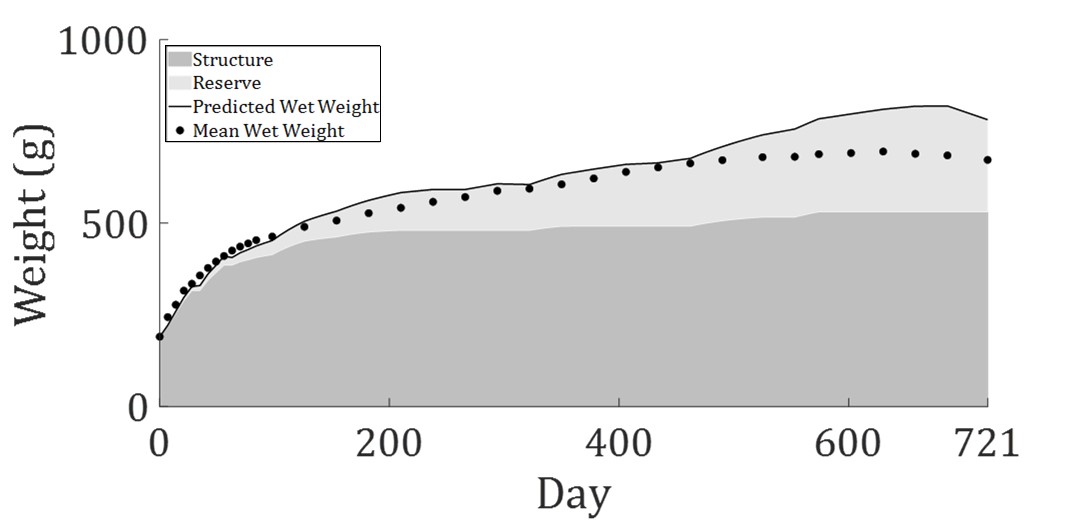


**Fig. S35** Observed (circles) and predicted (solid line) wet weight of male rats in group C over two years, using Method 3. The shaded area under the model curves shows structure (dark grey) and reserve (light grey).


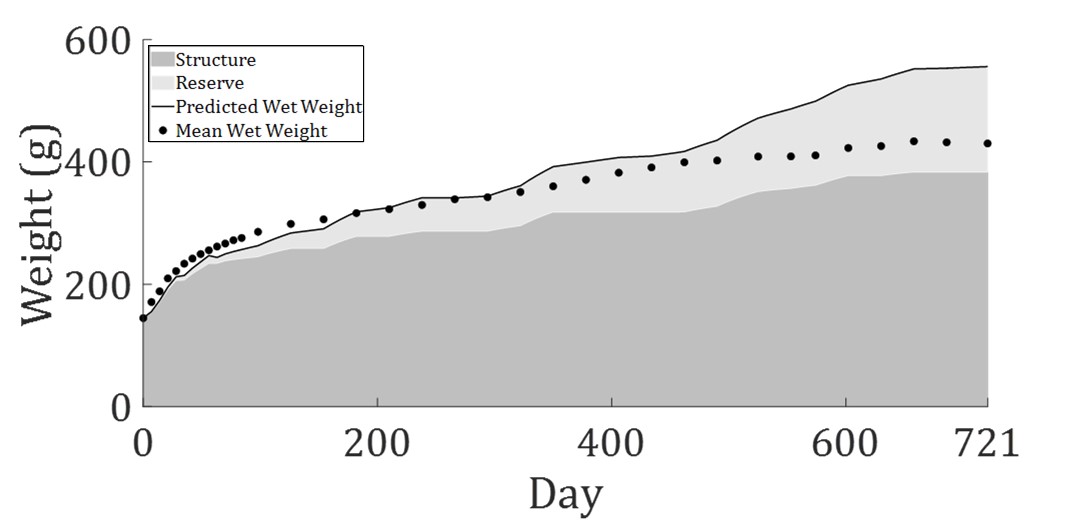


**Fig. S36** Observed (circles) and predicted (solid line) wet weight of female rats in group c over two years, using Method 3. The shaded area under the model curves shows structure (dark grey) and reserve (light grey).

**Feeding Data**


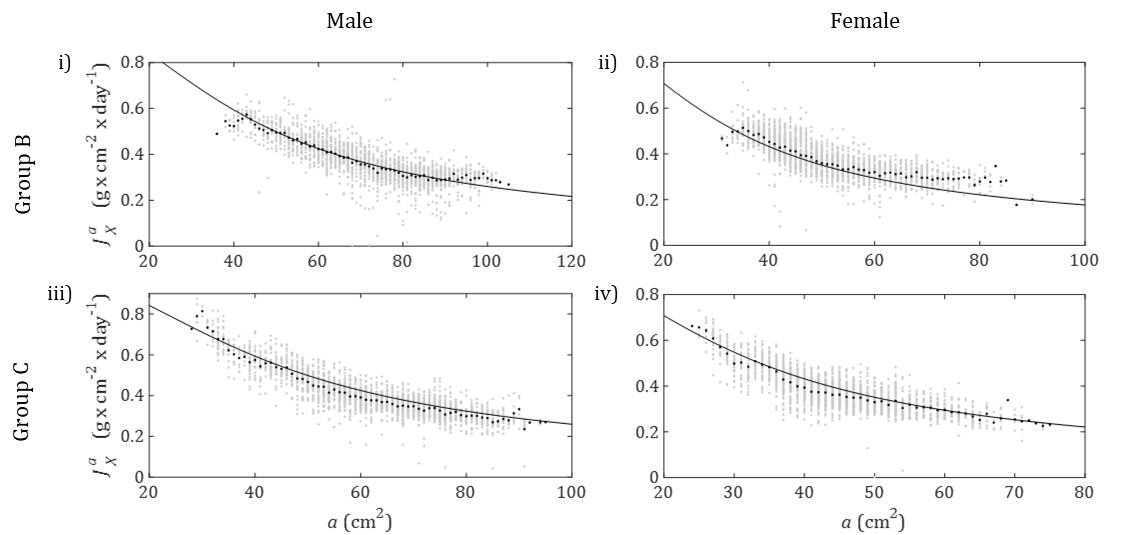


**Figure S37** Plots comparing independent data (circles) to predictions (lines) of area specific feeding rate, $J_{X}^{a}$ vs surface area, *a*. Raw data are plotted in light grey while mean values are plotted in black. Data for males in females in group B are shown in plots i & ii respectively while data for males in females in group C are shown in plots iii & iv respectively

**Figure S38** Observed area specific feeding rate, $J_{X}^{a}$, of male rats in group A plotted against surface area, *a*. Raw data are plotted in light grey while mean values are plotted in black. The dashed line shows the relationship required for scaled feeding rate, *f* = 1 if digestive efficiency, $y_{AX}$, is fixed as a primary parameter. Maximum area specific feeding rate, $J_{Xm}^{a}$, is assigned the value of the highest observation in the dataset.
