# Supplementary material for: Modelling the effects of variability in feeding rate on growth – a vital step for DEB-TKTD modelling
Source: Ecotoxicol Environ Saf. 2022 Mar 1;232:113231. doi: 10.1016/j.ecoenv.2022.113231 (PMC8873987; doi:10.1016/j.ecoenv.2022.113231)
Supplement: Supplementary file 2 — Supplementary material. [file mmc2.zip › Data in Brief.docx]

**Data in Brief for: Modelling effects of variability in feeding rate on growth – a vital step for DEB-TKTD modelling**

Thomas Martin^1🖂^ , Mark E Hodson^1^, Roman Ashauer^1,2^

1. University of York, Environment Department, Heslington, York, YO10 5NG, UK
2. Syngenta Crop Protection AG, Basel, 4002, Switzerland

🖂 [TM604@York.ac.uk](mailto:TM604@York.ac.uk)

**Guide to Raw Data File**

**Data**

The raw data come from the control groups in 2-year dietary toxicity studies. Data from males and females in three datasets (Groups A, B & C) are included. The full details of each dataset can be found in the ‘Methods’ section of the main manuscript.

**Excel File**

Body eight and food consumption data are included in an excel workbook, grouped into 12 sheets according to group (A, B or C) and sex (M/F).

Sheet names follow the formula ‘Group, Sex, Data Type (Units)’. For example body weight data for group A females in sheet ‘Grp A, F, Body Weight (g)’ while food consumption data for group C males are in sheet ‘Grp C, M, Feeding (g x week^-1)’.

**Timepoints**

The left-hand column contains the timepoint (in weeks from the beginning of the study) associated with data in each row. Body weight data were collected at the end of a study week. The one exception is the first measurement which took place at Week 0 (i.e. the beginning of Week 1).

Food consumption data (g × week^−1^) give the average rate of food consumption in the interval leading up to the timepoint in the left-hand column. For groups A and C food consumption was monitored in the week before the study began (Week -1) so there are feeding data at Week 0. For Group B, food consumption was not monitored during Week -1.

**Cage Number and Animal Number**

For groups A and C, food consumption data were collected per cage. For these datasets cage number is given in the top row, animal number is also given in row 2 alongside body weight data. For group B animals were monitored individually so only animal number is given.

**Empty Cells**

Data are not included at every timepoint for every rat. Naturally, data collection was ended if a rat died or was removed from the study. In some cases, data were not included for other reasons. For example, because a rat was dehydrated when weighed or because the food bowl had been spilled.
